# Critical assessment of wheat biofortification for iron and zinc: a comprehensive review of conceptualization, trends, approaches, bioavailability, health impact, and policy framework

**DOI:** 10.3389/fnut.2023.1310020

**Published:** 2024-01-04

**Authors:** Om Prakash Gupta, Ajeet Singh, Vanita Pandey, Ramadas Sendhil, Mohd. Kamran Khan, Anamika Pandey, Sunil Kumar, Mehmet Hamurcu, Sewa Ram, Gyanendra Singh

**Affiliations:** ^1^Division of Quality and Basic Sciences, ICAR-Indian Institute of Wheat and Barley Research, Karnal, Haryana, India; ^2^Division of Social Sciences, ICAR-Indian Institute of Wheat and Barley Research, Karnal, Haryana, India; ^3^Department of Soil Science and Plant Nutrition, Selcuk University, Konya, Türkiye

**Keywords:** hidden hunger, Fe and Zn content, phytic acid and phytase, bio-fortification, bioavailability, anti-nutrition factor, micronutrient deficiency

## Abstract

Addressing global hidden hunger, particularly in women of childbearing age and children under five, presents a significant challenge, with a focus on iron (Fe) and zinc (Zn) deficiency. Wheat, a staple crop in the developing world, is crucial for addressing this issue through biofortification efforts. While extensive research has explored various approaches to enhance Fe and Zn content in wheat, there remains a scarcity of comprehensive data on their bioavailability and impact on human and animal health. This systematic review examines the latest trends in wheat biofortification approaches, assesses bioavailability, evaluates the effects of biofortified wheat on health outcomes in humans and animals, and analyzes global policy frameworks. Additionally, a meta-analysis of *per capita* daily Fe and Zn intake from average wheat consumption was conducted. Notably, breeding-based approaches have led to the release of 40 biofortified wheat varieties for commercial cultivation in India, Pakistan, Bangladesh, Mexico, Bolivia, and Nepal, but this progress has overlooked Africa, a particularly vulnerable continent. Despite these advancements, there is a critical need for large-scale systematic investigations into the nutritional impact of biofortified wheat, indicating a crucial area for future research. This article can serve as a valuable resource for multidisciplinary researchers engaged in wheat biofortification, aiding in the refinement of ongoing and future strategies to achieve the Sustainable Development Goal of eradicating hunger and malnutrition by 2030.

## 1 Introduction

### 1.1 Snippets of global hunger and malnutrition

**Figure 1:**
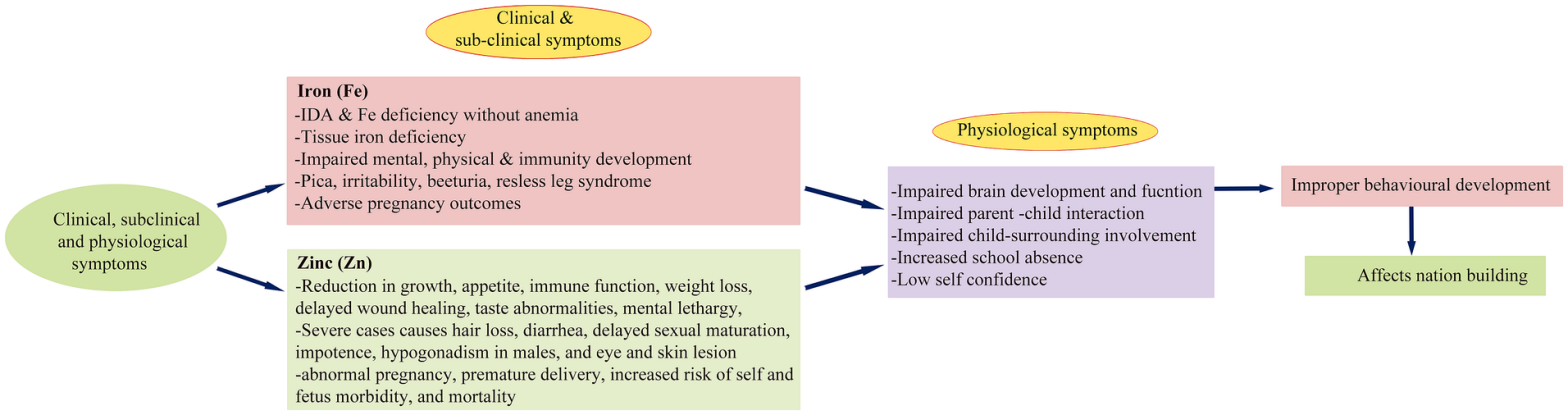
Schematic representation of clinical, sub-clinical and physiological deficiency symptoms of Fe and Zn in children and pregnant women and their impact on nation-building.

Fe and Zn deficiencies are the most widespread micronutrient deficiencies (MND) globally, affecting pregnant women (38%) and children under 5 years old (43%), akin to the enduring issue of poverty (FAO, 2019). Micronutrients are essential, as the body cannot synthesize them, making diet the sole source of these nutrients. Despite the critical importance of adequate dietary Zn, 17.3% of the global population, particularly pregnant women and children, still fall short of recommended Zn intake levels (1). Inadequate Zn intake, as per recommended dietary allowances (RDA), results in severe clinical, sub-clinical, and physiological symptoms in these vulnerable groups (Figure 1). Globally, 21.3% (144 million) of children under five suffer from malnutrition-induced stunting, with the highest prevalence observed in Africa (29.1%; 57.5 million; Supplementary Table S1) (2). Studies indicate a positive correlation between regions with high stunting rates (>20%), such as Sub-Saharan Africa and South Asia, and elevated levels of inadequate Zn intake. Despite a noteworthy reduction in global under-five mortality, declining from 93 deaths per 1000 live births in 1990 to 39 in 2018, Sub-Saharan Africa still grapples with elevated under-five mortality rates, reaching 78 deaths per 1000 live births in 2018, down from 182 in 1990 (3).

Anemia exerts detrimental effects on cognitive and motor development, causing fatigue, impairing mental and physical faculties, and compromising immune system development (Figure 1). Globally, approximately 38% of pregnant women experience anemia during various stages of pregnancy (4), characterized by a decrease in hemoglobin levels (to 120 g/L) in the blood (WHO, 2011). Regions such as Africa, South-East Asia, and the Eastern Mediterranean have reported the highest anemia prevalence, reaching 35% (Supplementary Figure S1) (5). It is, however, perplexing to observe only marginal improvements in global mean hemoglobin levels between 1995 and 2011 in non-pregnant women (from 125 to 126 g/L), pregnant women (from 112 to 114 g/L), and children (from 109 to 111 g/L). This modest increase has led to a reduction in anemia incidence in non-pregnant individuals (from 33 to 29%), pregnant women (from 43 to 38%), and children (from 47 to 43%). Nevertheless, this still translates to a substantial number of affected individuals, approximately 496 million non-pregnant, 32 million pregnant, and 273 million children in 2011 (4). Despite these marginal improvements, the persistently high numbers of affected individuals underscore the imperative for focused interventions aligned with RDA (Supplementary Table S2). Failure to do so may result in an additional 265 million anemic women by 2025 (6).

Securing adequate nutrition and ensuring robust health are inherent human rights and pivotal components of effective human capital development. Nevertheless, a critical examination of existing research data leads us to postulate that prevailing efforts aimed at mitigating global hunger and malnutrition, particularly concerning Fe and Zn deficiencies, are insufficient. This raises substantial concerns and formidable obstacles in achieving the United Nations’ Sustainable Development Goals (SDGs) of eradicating hunger and malnutrition by 2030. Therefore, it becomes imperative, and indeed, a pressing necessity, to foster worldwide collaboration and cooperation among crucial organizations. This concerted effort should encompass diverse strategies, encompassing dietary interventions and supplementation, to combat Micronutrient Deficiency (MND) on a global scale. Within this framework, following section elucidates why wheat and its biofortification represent a cost-effective and sustainable approach toward alleviating the burden of Fe and Zn-related MND, particularly in low and middle-income countries.

### 1.2 Analyzing wheat’s contribution to global nutrition: a meta-analysis of macronutrient and MNs provision

**Figure 2:**
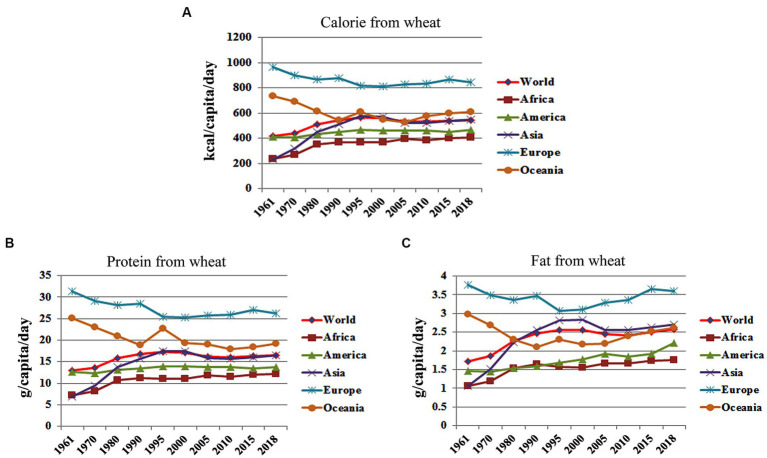
Changes in global consumption pattern of calorie, protein and fat from wheat between 1961 to 2018. (Data Source: FAOSTAT, New Food Balance Sheet, 2020). **(A)** Calorie from wheat; **(B)** Protein from Wheat; **(C)** Fat from wheat.

Wheat, with a global production of 790.6 million tonnes in 2022–23, stands as the second most prominent crop worldwide, trailing only maize, which yielded 1155.6 million tonnes (7).^1^ To underscore the pivotal nutritional role of wheat in the human diet on a global scale, a meticulous meta-analysis was conducted to evaluate its contribution to daily macronutrient (protein, energy, and fat) and micronutrient (Fe and Zn) intake, based on recent food balance sheet data (8),^2^ spanning from 1961 to 2018. The analysis unveiled that wheat plays a fundamental role in meeting daily caloric requirements, with the global populace consuming 544 kcal/person/day in 2018, accounting for 23.8% (Figure 2A) of the estimated daily caloric requirement of 2285 kcal/person/day (9). Additionally, for protein, with the recommended daily allowance (RDA) set at 0.8 g/kg body weight/day (10) and the average global population weight estimated at 62 kg, wheat supplied 16.4 g/capita/day of protein in 2018 (Figure 2B), covering 33.06% of the daily protein requirements for the global population. Furthermore, wheat contributed 3.3% of daily fat intake, with a global consumption of 2.58 g/capita/day in 2018 (Figure 2C), while the *per capita* fat supply globally increased from 48 grams in 1961 to 83 grams in 2014. Regional disparities were noted, with Europe and Oceania surpassing the global averages for caloric and protein consumption, while Africa exhibited the lowest intake levels (Figures 2A,B), potentially attributed to differing dietary habits and lifestyles, particularly influenced by Western dietary patterns.

**Figure 3:**
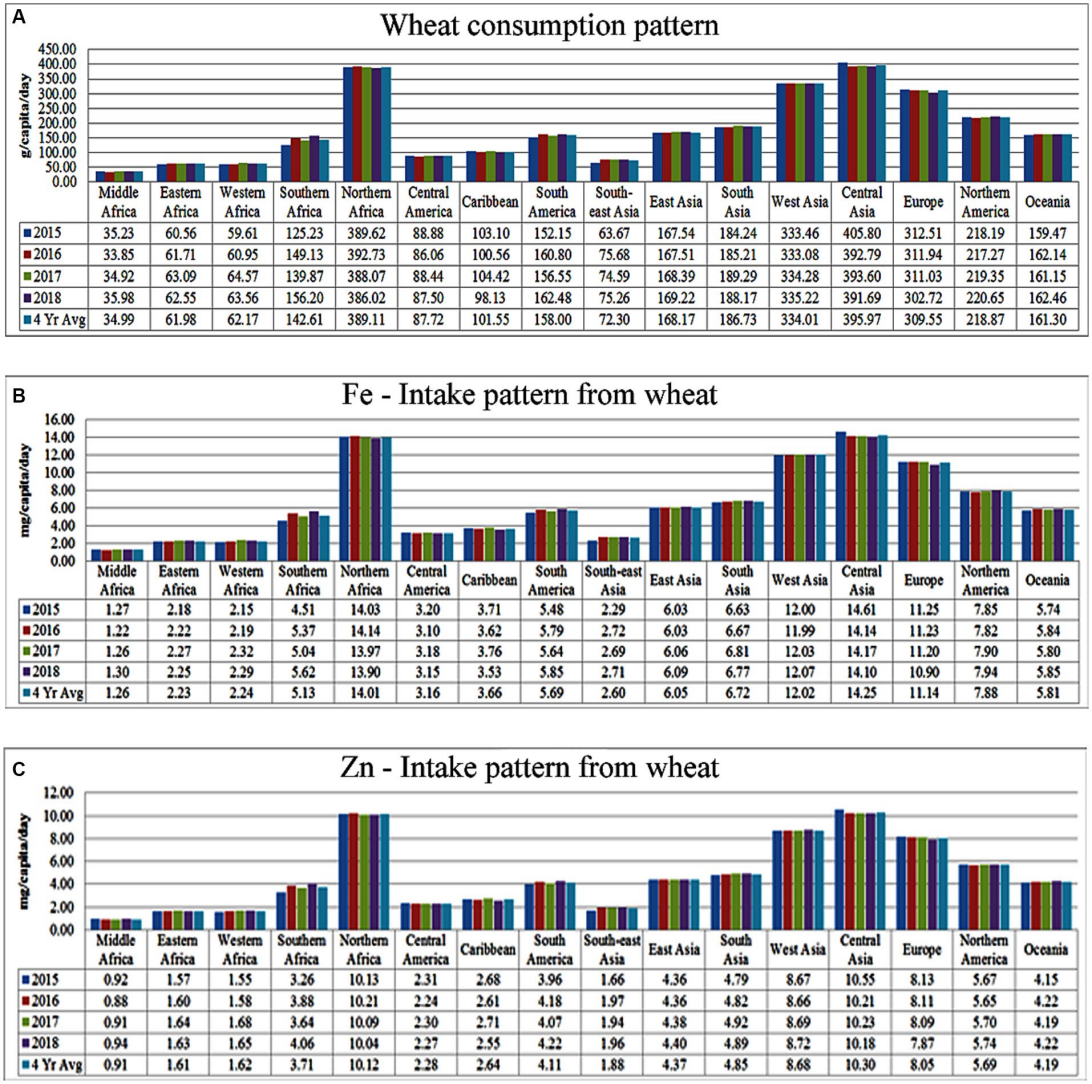
Status of region-specific Fe and Zn intake from wheat consumption. **(A)** *Per capita* wheat consumption; **(B)** Fe; and **(C)** Zn intake from wheatconsumption. (Primary Data Source: FAOSTAT, 2020).

A meta-analysis was conducted to ascertain the average wheat consumption patterns across different global regions and sub-regions during the period from 2015 to 2018 (Figure 3A). To accomplish this, primary data pertaining to the total wheat food balance for the years 2015 to 2018 were acquired from the new food balance sheet (FAOSTAT, 2020), along with the respective total population figures for each year. Subsequently, the *per capita* daily wheat consumption for each region was computed by dividing the total food balance by the population of that region. The four-year average revealed that global wheat consumption averaged approximately 180 grams per day, with notably lower consumption in developing countries, particularly in middle Africa (~34.99 g), followed by Eastern Africa (61.98 g) and Western Africa (62.17 g; Figure 3A). Furthermore, utilizing the region-specific wheat consumption patterns, an estimation was made of the contribution of wheat to total Fe (Figure 3B) and Zn (Figure 3C) intake. This estimation was based on standard reference values for Fe (36 mg kg-1) and Zn (26 mg kg-1) content in wheat grain as reported by the US Department of Agriculture, Agricultural Research Service (11).^3^ The results demonstrated that the consumption trends of these essential micronutrients (MNs) mirrored the overall wheat consumption patterns (Figures 3B,C). Global wheat consumption provides an average daily intake of 6.49 mg of Fe and 4.69 mg of Zn *per capita*. Notably, Middle Africa exhibits the lowest intake levels, with only 1.26 mg of Fe and 0.91 mg of Zn, followed by Eastern Africa (Fe: 2.23 mg; Zn: 1.61 mg) and Western Africa (Fe: 2.24 mg; Zn: 1.62 mg).

This data suggests that despite wheat’s prominence in the diets of many, especially in developing regions, it falls short of meeting recommended daily allowances (RDAs) for Fe and Zn. This inadequacy is primarily attributed to low levels of Fe and Zn in wheat grains and an unfavorable phytic acid (PA) to phytase ratio, limiting mineral bioavailability. To address this issue, there is a need for a pragmatic, time-bound strategy to develop biofortified wheat varieties with improved Fe and Zn content, enhancing their bioavailability. Simultaneously, strengthening public distribution systems is crucial to ensure timely access to these nutrient-rich wheat varieties for the world’s vulnerable populations.

### 1.3 Biofortification: an efficient, cost-effective, and sustainable approach to reach the unreached, outperforming fortification

Fortification, an ancient practice, stands out among various food-based strategies like diversification and supplementation as a rapid and effective means to combat micronutrient (MNs) deficiencies. Wheat flour, being a dietary staple, is a preferred vehicle for fortification, especially for essential micronutrients like Fe, Zn, and calcium (Ca). Supplementary Table S3 presents a global chronicle of wheat flour fortification trends with MNs and vitamins. Research indicates that fortification has indeed enhanced the presence of nutrients like iodine (I), Fe, folate, and vitamin A in many regions worldwide. However, more than 80 developing countries, spanning low, middle, and upper-middle-income categories, have not embraced fortification in any potential food vehicles, including wheat flour. In contrast, approximately 85 other countries have established mandatory wheat flour fortification programs with effective implementation (12–14).^4^ It is noteworthy that fortified flour may undergo various physiochemical changes and alterations in physical and sensory attributes, potentially affecting consumer acceptability.

Conversely, bio-fortification has emerged as an efficient, sustainable and cost-effective way to ameliorate MNs associated health outcomes with reduced mortality and morbidity in resource-poor and developing nations. Owing to its inherent superiorities over fortification (Supplementary Table S4) (15, 16), it has broader applicability in disseminating to the nutritionally deprived regions, especially Africa and Asia. Several bio-fortified crops, including orange, sweet potatoes, maize, cassava, squash, Fe-enriched beans, sorghum, lentils, pearl millet, and Zn-enriched rice, and wheat etc. have been released in different parts of the world, catering for a range of MNs for the people who do not have access to other interventions (17). For every dollar invested in bio-fortification, as much as US$17 of benefits may be gained sustainably, which is far better than the fortification strategy (18). In addition, once bio-fortified wheat cultivars are under farmer’s field, recurrent expenditures for monitoring and maintenance are minimal, and after fulfilling the household’s requirements, the surplus can enter into different retail outlets situated in urban and suburban areas. As wheat, serves as a primary staple food across the world (19, 20) and is consumed by ~2.5 billion people from around 89 countries, we have comprehensively reviewed and hypothesized the global progress made on different dimensions of wheat bio-fortification, including status and approaches, anti-nutritional factors and bioavailability, advances in assay methods, proof-of-concept from animal and human trials followed by a policy framework to strengthen the bio-fortification value chain.

## 2 Present strategies and progress in wheat biofortification for Fe and Zn

### 2.1 Agronomic approach via soil and foliar application of Fe and Zn

In recent decades, agronomic biofortification, specifically ferti-fortification, has emerged as a highly efficient method for enhancing MNs levels, such as Zn and Fe, in wheat crops. This approach has garnered significant attention due to its effectiveness and speed in elevating Zn and Fe content in wheat grains. Several studies have confirmed the efficacy of agronomic biofortification in augmenting Zn and Fe levels in wheat grain (21). Notably, the application of different Fe sources via foliar application has demonstrated a positive correlation with increased Fe content in wheat grain (22). The genetic diversity among various wheat genotypes has emerged as a key determinant influencing the efficiency of foliar application-based enrichment of Fe and Zn in wheat grain. Moreover, the simultaneous foliar application of multiple MNs, including Fe and Zn, has led to a substantial improvement in flour Fe (22%) and Zn (21%) across diverse wheat lines (23). This approach, encompassing a mix of multiple MNs such as Fe, Zn, selenium (Se), and iodine (I), is gaining prominence over traditional single MN-based foliar application due to its potential advantages. Furthermore, aside from enhancing grain Fe and Zn content, foliar application of Fe and Zn has exhibited significant enhancements in grain yield and protein content (24). The underlying mechanism responsible for these advantages is not yet fully understood but may be associated with heightened enzymatic activity, leading to increased photosynthesis and enhanced translocation of assimilates to the seed. To confirm this hypothesis, large-scale randomized field trials involving a diverse genetic pool of wheat are warranted.

**Figure 4:**
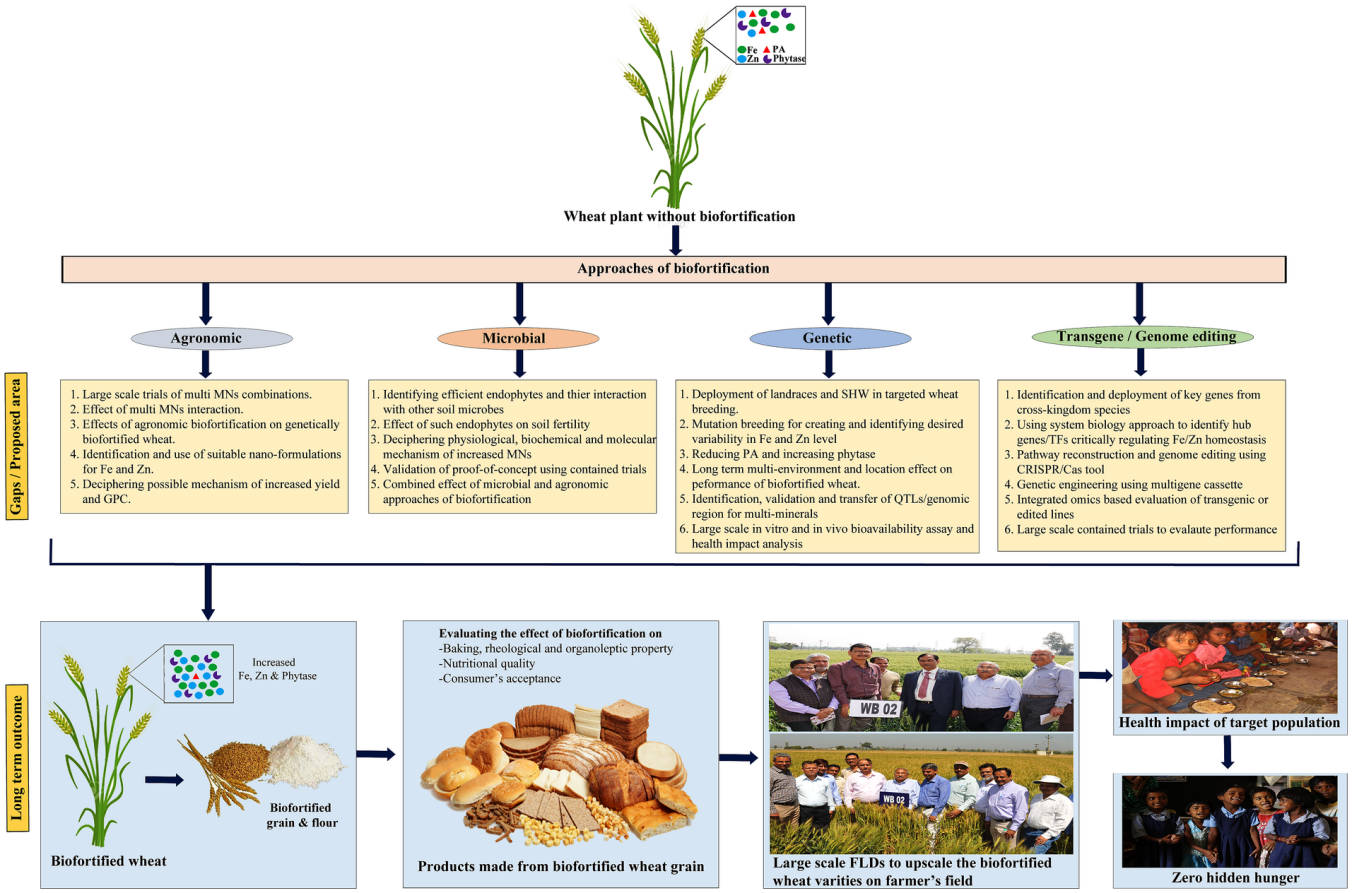
Schematic working model with identified gaps/proposed areas of future intervention along with long-term outcomes for a targeted wheat bio-fortification program to ameliorate the MNM globally. PA, Phytic acid; QTL, Quantitative trait loci; CRISPR/Cas, clustered regularly interspaced short palindromic repeats/CRISPR associated protein; FLD, Front-line demonstration.

An emerging area of interest involves assessing the potential of soil or foliar application of Fe and Zn to further enhance the MN levels in recently developed Fe and Zn biofortified wheat varieties. A recent study conducted by (25) demonstrated this hypothesis. They reported a substantial increase of approximately 43% in the total Zn content, reaching 53 mg kg^−1^, and an enhancement in Zn bioavailability to 2.8 day^−1^ in a Zn-biofortified wheat cultivar known as Zincol-2016, originally containing 37 mg Zn kg^−1^, through the application of Zn to the soil at a rate of 6 mg kg^−1^. Nevertheless, further research is warranted to assess the reliability and practical applicability of agronomic biofortification methods in genetically biofortified wheat cultivars. The utilization of nano-fertilizers, particularly nano-coated urea, is gaining popularity among farmers. However, limited reports are available regarding the use of Fe and Zn complexed nanoparticles as a viable medium for agronomic biofortification. An endeavor by (26) sought to illustrate the effectiveness of employing Zn complexed chitosan nanoparticles (Zn-CNP) for ferti-fortification of durum wheat in field-scale experiments. The results demonstrated that Zn-CNP led to a approximately 36% increase in Zn content, which was comparable to the conventional use of ZnSO_4_, despite using a significantly lower concentration of Zn(40 mg L^−1^) in the former. Although still in its nascent stages, the utilization of Fe and Zn complexed nanoparticles has introduced a novel dimension to agronomic biofortification in wheat grains. However, comprehensive research is imperative to evaluate its influence on baking, rheological, and nutritional qualities of wheat-based products, aiming to facilitate its widespread adoption by both farmers and consumers (Figure 4).

In summary, several crucial factors, including soil characteristics, genetic variability within wheat varieties, the composition of applied fertilizers, and their potential interactions, significantly influence the variability observed in the Fe and Zn content of wheat grains. In comparison to soil-based application methods, the foliar application of Fe and Zn appears to be more effective due to its efficient uptake by plant foliage, without the risk of soil immobilization. These findings highlight the substantial potential of agronomic biofortification as a rapid intervention to enhance Fe and Zn levels in wheat grains. This strategy holds promise for swiftly mitigating the global risk of MNDs, particularly in resource-constrained populations, offering a viable approach to addressing this pressing public health issue.

### 2.2 Enhancing nutrient content via microbiota-driven plant growth promotion

The accumulation of research in understanding the interactions between soil, microorganisms, and plants has significantly advanced our understanding of the potential role of microorganisms in combination with fertilization as a promising strategy for enriching micronutrients (MNs) in crop grains. Over several decades, rhizospheric and endophytic microorganisms have been recognized for their ability to solubilize metals in the soil, thereby facilitating the redistribution of MNs within plants, including the grains. Existing studies have reported the beneficial impact of various microorganisms on increasing the Fe and Zn content in wheat grains (27–29). In general, both rhizospheric and endophytic microbes play crucial roles in enhancing MN availability to plants due to their metal-solubilizing properties. However, endophytic microbes exhibit greater promise in improving the uptake and transport of Fe and Zn to various plant tissues. This is attributed to their significant capacity to modulate the expression of various metal transporters, including those responsible for the uptake of Fe and Zn.

A comprehensive review of literature on the use of microbes in Fe and Zn bio-fortification and their significant outcomes in wheat grain have been enlisted in Table 1. The predominant microbial categories employed by researchers include rhizospheric and endophytic microbes, exhibiting diverse capabilities such as Fe and Zn solubilization and the synthesis of phytosiderophores (PS). While the precise mechanisms underlying microbe-mediated increases in Fe and Zn content in wheat grains remain poorly elucidated, potential mechanisms have been proposed. These mechanisms include the secretion of PS, organic acids, phenolic compounds, phytohormones, alterations in root morphology, up-regulation of Fe and Zn transporters, and reduction of antinutritional factors like PA (19, 40). However, these proposed mechanisms require further validation for their practicality and potential applicability in large-scale trials, including on farmers’ fields, as depicted in Figure 4. Moreover, there is scope for additional research efforts to identify novel and efficient sources of rhizospheric or endophytic microbes, delving into their physiological, molecular, and biochemical basis for enhanced solubilization of various metals, including Fe and Zn in soil and their subsequent transportation to wheat grains. Microbe-based wheat biofortification holds substantial promise for enhancing MN levels, including Fe and Zn, while simultaneously improving soil fertility and crop yield. This approach serves as a valuable supplement to conventional strategies like breeding and fertilization. Furthermore, it presents an environmentally friendly alternative, potentially mitigating the adverse health consequences associated with extensive chemical fertilizer, pesticide, and agrochemical use.

**Table 1:** List of microbes utilized for wheat grain bio-fortification with Fe and Zn.

Name of the microbes	Nature of microbes	Micronutrient bio-fortified	Significant outcomes	References
PGPR- AW1 *Bacillus* sp.; AW5 *Providencia* sp.; AW7 *Brevundimonas* sp. as individual and in combinations	Plant growth-promoting rhizobacteria	Fe, Zn, Cu, Mn	Increased micronutrient content 28–60% with AW1 + AW5 treatment	Rana et al. (30)
*Providencia* sp. PW5	Bacteria	Fe, Cu, Mn	PW5 significantly increased Fe (105.3%), Mn (36.7%), and Cu (150.0%)	Rana et al. (31)
CW1 *Anabaena laxa*, CW2 *Calothrix* sp., and CW3 *Anabaena* sp	Cyanobacteria			
*Bacillus aryabhattai* strains MDSR7, MDSR11 and MDSR14	Zn solubilizing	Zn	Substantially influenced mobilization of zinc and its concentration in the edible portion	Ramesh et al. (32)
*Pseudomonas fluorescens* strain Psd	Plant growth-promoting rhizobacteria	Zn	Grain Zn content was enhanced by ~85% in comparison to wheat grown in Zn deficient soil	Sirohi et al. (33)
*Serratia liquefaciens*, *S. marcescens* and *Bacillus thuringiensis*	Plant growth-promoting rhizobacteria	Zn	Improved grain yield and Zn content	Abaid-Ullah et al. (34)
*Bacillus subtilis* DS-178 and *Arthrobacter* sp. DS-179	Zn solubilizing	Zn	Enhanced translocation and enrichment of Zn to grains	Singh et al. (35)
*Arthrobacter sulfonivorans* DS-68	Siderophore producing	Zn & Fe	1.4 fold increase in shoot Zn & Fe, and up-regulation of *TaZIP3* and *TaZIP7* genes in roots and shoots	Singh et al. (36)
*Arthrobacter* sp. DS-179	Zn solubilizing			
*Pseudomonas* sp. MN12	Zn solubilizing	Zn	Reduced molar ration of [phytate]: [Zn] and increased the bioavailable Zn	Rehman et al. (37)
*Bacillus subtilis* DS-178 and *Arthrobacter* sp. DS-179	Zn solubilizing	Fe and Zn	1.5 fold increase in Fe and Zn content	Singh et al. (38)
*Arthrobacter sulfonivorans* DS-68 and *Enterococcus hirae* DS-163	Siderophore producing			
*Arthrobacter sulfonivorans* DS-68 and *Enterococcus hirae* DS-163	siderophore-producing endophytes	Fe	Increase of 1.5-fold and 2.2-fold in grain Fe content over the RDF + FeSO_4_ treatment and uninoculated control (RDF), respectively	Singh et al. (39)
*Anabaena* sp. (CR1) + *Providencia* sp. (PR3) consortium and *Anabaena*–*Pseudomonas* (An-Ps)	Cyanobacteria	Fe	Increased total Fe uptake by 133–151 g ha^−1^	Shahane et al. (28)

### 2.3 Genetic approach: conventional and molecular breeding

Genetic strategies, encompassing both conventional and molecular breeding techniques, are widely regarded as effective methods for enhancing Fe and Zn biofortification in crops, including wheat. Conventional breeding involves leveraging existing genetic diversity within the primary, secondary, and tertiary gene pools to augment the Fe and Zn content in wheat grains. Consequently, an essential prerequisite for elevating Fe and Zn levels in wheat grains is the presence of genetic diversity in the targeted traits. In contrast, mutation breeding employs physical and chemical mutagens, as well as induced local lesions in genomes (TILLING), to introduce variability in traits where limited or no natural variations exist. Additionally, the molecular breeding approach incorporates the identification of quantitative trait loci (QTLs) and marker-trait associations (MTAs) using marker-assisted selection (MAS), marker-assisted recurrent selection (MARS), and genomic selection (GS). However, it is worth noting that the success of these molecular breeding techniques has been somewhat constrained, as discussed in the latter part of this section.

Available scientific literature suggests that, when compared to cultivated wheat varieties, wild, synthetic, and primitive wheat lines are valuable genetic resources for enhancing the Fe and Zn content in wheat grains (41, 42). Notably, diploid progenitors of hexaploid wheat, such as *Aegilops tauschii*, einkorn (*Triticum monococcum*), wild emmer (*T. dicoccoides*), *T. polonicum, T. spelta*, and landraces of *T. aestivum*, have shown promise as high Fe and Zn sources. Among the wild wheat varieties examined, collections of *Triticum turgidum* ssp. *dicoccoides* have demonstrated significant genetic variation, with Zn levels ranging from 14 to 190 mg kg^−1^ and Fe levels up to 88 mg kg^−1^ (43). Translocations from rye and various *Aegilops* spp. into the Pavon 76 wheat background, followed by utilization in breeding efforts at the International Maize and Wheat Improvement Center (CIMMYT, Mexico), involving landraces, recreated synthetic hexaploids, *T. spelta*, and several pre-breeding lines, have resulted in wheat lines with variable Zn content in grains, ranging from 35 to 69 mg kg^−1^ in 2017 and 38 to 72 mg kg^−1^ in 2018. Conversely, grain Fe content exhibited smaller fluctuations, ranging from 30 to 43 mg kg^−1^ in 2017 and 32 to 52 mg kg^−1^ in 2018. Furthermore, a significant positive correlation between Fe and Zn content (*r* = 0.54; *p* < 0.001) has been observed, enabling breeders to concurrently enhance both elements (41). In addition, the potential of synthetic hexaploid wheat (SHW) to improve grain nutrient content has been actively pursued by CIMMYT, with the development of over 1600 SHWs integrated into large-scale breeding programs aimed at expanding genetic diversity and enhancing MN levels (44). Through collaborative efforts involving CIMMYT wheat breeders and major wheat institutions in India, Pakistan, Bangladesh, and Bolivia, a total of forty biofortified wheat varieties have been developed and released for commercial cultivation in their respective regions (15, 45) (Table 2). Genetic and agronomic approaches have garnered greater global acceptance compared to other methods and have therefore become the primary research focus (46). The incorporation of wild relatives and synthetic hexaploid wheat (SHW) in conventional breeding programs has demonstrated promise for the development of biofortified wheat varieties enriched in micronutrients (MNs). Despite these advancements, several critical and unanswered questions emerge for future research (Figure 4), which could enhance the effectiveness of wheat biofortification programs. These questions pertain to the long-term performance of biofortified varieties across diverse environments and locations. Key parameters to consider include the stability of Fe and Zn content, yield, end-product quality, *in vitro* and *in vivo* bioavailability, as well as the health impact on the target population. Addressing these inquiries will contribute to the continued success and expansion of biofortified wheat initiatives.

**Table 2:** List of bio-fortified wheat varieties developed by conventional breeding and released for commercial cultivation around the globe [Adopted from Gupta et al. (15)].

Variety	Nutritional quality	Other features	Year of release	Developer/sources
India				
WB 02	Zn: 42; Fe: 40	Yield: 51.6 q/ha	2017	ICAR-Indian Institute of Wheat and Barley Research, Karnal, India
DBW 173	Fe: 40.7 Protein: 12.5	Yield: 47.2 q/ha	2018	
DBW 187 (Karan Vandana)	Fe: 43.1	Yield: 48.8 q/ha (NEPZ),61.3q/ha (NWPZ), 75.5 q/ha (High fertility)	2018 and 2020	
DDW 47	Fe: 40.1 Protein: 12.7	Yield: 37.3 q/ha	2020	
DBW 303	Fe: 35.8; Zn: 36.9; Protein: 12.1	Yield: 81.2 q/ha	2020	
DDW 48	Fe: 38.8; Zn: 39.7; Protein: 12.1	Yield: 47.4 q/ha	2020	
HPBW 01 (Ankur Shiva)	Zn: 40.6; Fe: 40	Yield: 51.7 q/ha	2017	Punjab Agricultural University (PAU), Ludhiana, India
PBW 752	Fe: 37.1; Zn: 38.7; Protein: 12.4	Yield: 49.7 q/ha	2018	
PBW 757	Zn: 42.3	Yield: 36.7 q/ha	2018	
PBW 771	Zn: 41.4	Yield: 50.3 q/ha	2020	
HI 8759 (Pusa Tejas)	Zn: 42.8; Fe: 42.1; Protein: 12.0	Yield: 50.0 q/ha	2017	ICAR- Indian Agricultural Research Institute, Regional Station, Indore, India
HI 1605 (Pusa Ujala)	Zn: 35; Fe: 43 Protein: 13	Yield: 30.0 q/ha	2017	
HI 8777	Fe: 48.7; Zn: 43.6	Yield: 18.5 q/ha	2017	
HI 8802	Fe: 39.5; Zn: 35.9; Protein: 13.0	Yield: 29.1 q/ha	2020	
HI 8805	Fe: 40.4; Protein: 12.8	Yield: 30.4 q/ha	2020	
HI 1633	Fe: 41.6; Zn: 41.1; Protein: 12.4	Yield: 41.7 q/ha	2020	
HD 3171	Zn: 47.1	Yield: 28.0 q/ha	2017	ICAR- Indian Agricultural Research Institute, New Delhi, India
HD 3249	Fe: 42.5	Yield: 48.8 q/ha	2020	
HD 3298	Fe: 43.1; Protein:12.1	Yield: 43.7 q/ha	2020	
MACS 4028	Zn: 40.3; Fe: 46.1; Protein: 14.7	Yield: 19.3 q/ha	2018	Developed by Agharkar Research Institute, Pune, Maharashtra
MACS 4058	Fe: 39.5 Zn: 37.8 Protein: 14.7	Yield: 29.6 q/ha	2020	
UAS 375	Protein: 13.8	Yield: 21.4 q/ha	2018	University of Agricultural Sciences, Dharwad, India
BHU-1, Akshai (BHU-3), BHU-5, BHU-6, BHU-17, and BHU-18	High Zn	High yield, disease resistance	2014	Banaras Hindu University, Varanasi, India
Abhay (Zinc Shakthi)	High Zn	Registered by private seed companies and grower	2015	Nirmal Seeds, Harvest Plus and Participatory variety selection
Zinc Shakthi (Chitra)	High Zn	Registered by private seed companies and grower	2016	Participatory variety selection
Pakistan				
NR- 421 (Zincol-16)	High Zn	>6 ppm Zn advantage compared to best local check	2015	Pakistan Agriculture Research Council/CIMMYT
Akbar-19	High Zn	>7 ppm Zn advantage compared to best local check	2019	Faisalabad Agricultural Research Institute/CIMMYT
Bangladesh				
BARI Gom 33	High Zn	7–8 ppm Zn advantage over best check, and also resistance to wheat blast	2017	CIMMYT, Mexico
Mexico				
Nohely-F2018	High Zn	Nohely-F2018 released in Mexico for the Mexicali valley of northern Sonora region	2018	CIMMYT, Mexico
Bolivia				
Iniaf-Okinawa	High Zn	>6 ppm Zn advantage than the local check	2018	INIAF, Bolivia and CIMMYT, Mexico
Nepal				
Zinc Gahun 1	High Zn	>6 ppm Zn advantage than the local check	2020	NARC, Nepal and CIMMYT, Mexico
Zinc Gahun 2	High Zn	>6 ppm Zn advantage than the local check	2020	
Bheri-Ganga	High Zn	>6 ppm Zn advantage than the local check	2020	
Himganga	High Zn	>6 ppm Zn advantage than the local check	2020	
Khumal-Shakti	High Zn	>6 ppm Zn advantage than the local check	2020	

Grain Fe and Zn conents are expressed in ppm while protein content is expressed in percentage (%). NEPZ, Northern Eastern Plains Zone; NWPZ, Northern Western Plains Zone.

Advancements in computational analysis and cost-effective genome-wide molecular techniques, such as MAS, GS, and MARS, have facilitated the identification of numerous QTLs and genes with significant potential for enhancing the Fe and Zn content in wheat. These techniques leverage MTAs identified through QTL or association mapping to expedite trait selection during MAS (47). While MAS for multi-trait biofortification in wheat has commenced, its full potential remains untapped. In contrast, GS predicts the genetic value of individuals based on Genomic Estimated Breeding Values (GEBVs) derived from a dense set of markers spanning the entire genome. Compared to MAS, GS captures a larger portion of the genetic variation for the specific trait under selection by including markers with both minor and major effects. Numerous studies support the efficacy of GEBV-based approaches for wheat biofortification (48). Effective implementation of MAS necessitates updated knowledge of the genetic basis, including genes associated with efficient Fe and Zn uptake, translocation, and remobilization in wheat grains. Several QTL mapping studies have identified a collection of QTLs for both Fe and Zn in hexaploid wheat (49). Furthermore, various genes linked to the methionine cycle, phosphorus-sulfur biosynthesis, and transporters facilitating the effective uptake and mobilization of Fe and Zn from the rhizosphere to the grain have also been identified in recent research (50–52).

Drawing from the existing literature, we have identified critical research gaps and proposed key areas for future investigation (Figure 4). It is imperative to conduct in-depth research on the intricate role of epistatic interactions in influencing the expression of traits related to the accumulation of Fe and Zn in grains. Furthermore, there is a need to shift focus toward the identification of Quantitative Trait Loci (QTLs) and genomic regions associated with various minerals. This is warranted due to the shared involvement of these minerals in multiple biochemical and physiological pathways. However, it is also essential not to disregard genomic regions that regulate individual minerals, as they may play vital roles in mineral-specific mechanisms. Exploring the incorporation of genes from external sources and utilizing techniques such as induced homoeologous pairing through *ph1b* or monosomy for chromosome 5B, as well as irradiation for transferring alien genomic regions to elite plant lines, represents significant avenues for further research. These efforts aim to enhance both the quantity and bioavailability of grain Fe and Zn, particularly in monogastric animals.

### 2.4 Advances on transgenic approaches for enhancing Fe and Zn biofortification in wheat grains

While conventional breeding is globally accepted, the absence of desired genetic diversity for targeted traits within species (e.g., golden rice) or difficult to breed crop (e.g., banana) can efficiently be managed through genetic engineering technologies as a valid alternate. This technique offers limitless cross-kingdom utilization of desired genes for target trait improvement. Moreover, it offers simultaneous bio-fortification of multi nutrients by metabolic engineering (53). Knowledge gained in identifying and functional characterization of different genes actively associated with uptake, translocation, and storage of Fe and Zn can efficiently be used to increase their content in wheat using this approach. Several proofs of concepts using the genetic engineering approaches have been tested during the last decade with apparently interesting results in wheat for grain Fe and Zn. Here, we have critically evaluated the recent works during the past 5 years and surprisingly only a few effective pieces of work were observed. Two independent workers have depicted that the over-expression of *NICOTIANAMINE SYNTHASE2* (*OsNAS2*) gene in wheat produced Fe up to 93.1 mg kg^−1^ (36) and 80 mg kg^−1^ (54) under greenhouse and field conditions, respectively. Connorton et al. (55) demonstrated the doubling of the total Fe content in wheat flour by using *VACUOLAR IRON TRANSPORTER2* (*TaVIT2*) gene, which effectively enhances vacuolar Fe and manganese (Mn) transport in the endosperm. To increase Fe bioavailability, PA content was decreased by silencing the wheat *ABCC13* transporter gene (56). The scanty work in traditional transgene based approach for wheat bio-fortification might be associated with the issues of stability of different transgenes in subsequent generations, their large-scale field trials and, most importantly, the regulatory policy on transgenic plants globally. However, with the advent of powerful reverse genome editing tools especially clustered regularly interspaced short palindromic repeats/ CRISPR associated protein (CRISPR/Cas) along with publicly available wheat genome, several researchers across the globe have demonstrated its utility by altering various traits in wheat. Compared to the traditional transgenic approach, CRISPR/Cas tool is regarded safer and has more public acceptance globally; therefore, it could potentially be promising to increase multiple MNs content and their bioavailability by targeting genes associated with MNs uptake, translocation and storage in different tissues of wheat grain (Figure 4). This will increase the biochemical and physiological pathway’s efficiency, system biology (pathway reconstruction) and decrease the antinutritional factor to increase the bioavailability.

## 3 Progress in enhancing Fe and Zn bioavailability through research on adjusting phytic acid and phytase ratios

Bioavailability (amount of nutrients available for absorption), bioconversion (nutrients incorporated in biomolecules) and bioefficacy (sum of bioavailability and bioconversion) of nutrients, specifically Fe and Zn, are influenced by various factors. These include the Fe and Zn content of wheat genotypes, the ratio of PA to phytase, and host factors that impact the absorption, utilization, and excretion of these minerals in the human body. In cereals, particularly wheat, the bioavailability of Fe and Zn is generally low. This is attributed to variations in Fe and Zn levels among different wheat genotypes, as well as the presence of PA and other antinutritional factors that can reduce their bioavailability by up to 15% (57). PA, chemically known as myoinositol 1,2,3,4,5,6-hexakis dihydrogen phosphate, serves as the primary storage form of phosphorus (P) in cereals, accounting for 50–85% of total P (58). However, it acts as a significant anti-mineral factor due to its negative charges, which bind to various cations, including Fe and Zn, forming complexes known as PA (59). The presence of PA significantly diminishes the bioavailability of Fe and Zn in monogastric animals, including humans and poultry, primarily due to the absence of the PA-degrading enzyme called phytase. Additionally, the insolubility of PA-mineral complexes at physiological pH further contributes to the lower bioavailability of Fe and Zn (60).

Over time, the molar ratio of PA:Fe and PA:Zn has been recognized as a useful indicator for assessing the potential absorption and bioavailability of Fe and Zn, respectively. When assessing Zn bioavailability in wheat grains, the PA: Zn molar ratio can be categorized as follows: low (PA: Zn >15; bioavailability 10–15%), medium (PA: Zn 5–15; bioavailability 30–35%), and high (PA: Zn <5; bioavailability 45–55%) (61). Similarly, in evaluating Fe bioavailability, a PA: Fe molar ratio of <1 (preferably <0.4) in a plain diet consisting of either cereals or pulses without external enhancers is optimal for increasing Fe absorption. Additionally, the molar ratio and redox state of Fe play a crucial role in determining its bioavailability, with PA having a greater affinity for binding and chelating with Fe^3+^ compared to Fe^2+^, thereby reducing bioavailability (62). The development of low phytate (*lpa*) wheat lines with high phytase content, integrated into core-breeding programs, offers a viable model for enhancing Fe and Zn bioavailability in both humans and animals (Figure 4). It’s worth noting that improving Fe bioavailability from 5 to 20% is roughly equivalent to a four-fold increase in total Fe content. Consequently, genetic enhancement of Fe and Zn bioavailability is more achievable than achieving a similar increase in their total concentration. Among the factors influencing phytase and PA levels, genetic variability among wheat genotypes is the primary determinant. Non-lethal recessive *lpa* mutants have been successfully developed using various physical and chemical mutagens in cereal crops, including wheat (63). Furthermore, nicotianamine, a metal chelator molecule, has demonstrated the ability to enhance Fe and Zn bioavailability in Caco-2 cells (64). Research on the utilization of *lpa* mutants to transfer low PA traits to major wheat varieties, coupled with strategies to increase nicotianamine content in wheat endosperm through conventional, molecular, or genetic engineering approaches, is limited but holds potential as an emerging avenue for enhancing MNs bioavailability.

Dietary components like histidine, cysteine ligands, tripeptides, and endogenous ligands such as citric acid have been shown to enhance the absorption of Zn in the human body. Conversely, Fe and Zn absorption are both inhibited by substances with similar chemical properties, including PA, oxalate, and polyphenols. Developing dietary compositions with a higher ratio of enhancers to inhibitors is a promising avenue to enhance mineral bioavailability. Altering the levels of enhancer substances in plants is relatively straightforward since it involves only a few regulating genes, in contrast to breeding for higher Fe and Zn levels, which is a complex process influenced by numerous genes and environmental factors. While limited reports suggest that food-processing methods like Maillard reactions and heat treatment can create Zn complexes resistant to hydrolysis, thus affecting its absorption (65), further research is needed to confirm the inhibitory effect of elevated temperatures on mineral absorption and provide meaningful recommendations. Promoting practices such as soaking and fermentation may significantly improve mineral absorption as they partially hydrolyze PA into metabolites with reduced Zn-binding capacity and lower inhibitory effects. Taking together, it is advisable for food scientists, wheat breeders, and molecular biologists to collaborate in developing strategies that manipulate dietary composition and enhance Fe, Zn, and phytase levels while reducing PA content. This can be achieved through genetic enhancement using tools like CRISPR/Cas9 to maximize mineral availability for specific global populations.

## 4 Effect of biofortification on the spatial distribution of Fe and Zn in grain

While the total concentrations of Fe and Zn in wheat are significant, their effectiveness as a mineral source is not solely determined by quantity but also hinges on their bioavailability. Bioavailability, in this context, refers to the extent to which these minerals can be absorbed and utilized by the human body. Understanding the bioavailability of Fe and Zn in wheat grains is crucial for addressing widespread micronutrient deficiencies. This review delves into the intricate dynamics of mineral bioavailability in wheat and how genetic engineering offers a promising solution to enhance the nutritional quality of this dietary staple. In mature wheat grains, Fe and Zn exhibit a distinct spatial distribution. They are predominantly concentrated in two regions: the aleurone layer and the embryo. These regions house the bulk of these essential minerals, with limited amounts found in the starchy endosperm, which is the primary component of white flour (66). This unique distribution is pivotal in shaping the bioavailability of these minerals.

The aleurone layer and embryo of wheat grains contain discrete entities known as phytin globoids. These globoids are formed through the complexation of Fe and Zn with phytic acid, specifically inositol hexakisphosphate, resulting in low solubility (67). This low solubility presents a significant challenge for mineral bioavailability as it restricts the release and uptake of these minerals in the human gastrointestinal tract. Studies estimate that only about 25% of the Zn and 10% of the Fe present in wholegrain wheat are bioavailable. This limited bioavailability is primarily attributed to the prevalence of phytate salts in the aleurone layer and scutellum, rendering the minerals poorly soluble. This reduced solubility inhibits their accessibility to Fe transporters in the human gastrointestinal tract, thus limiting their absorption (68). This phenomenon underscores the need for strategies to enhance the bioavailability of these essential minerals, especially in regions where wheat is a dietary staple. Interestingly, the bioavailability of Zn and Fe in wheat exhibits a notable discrepancy. Zn, present in the embryonic axis, is associated with enzymes and proteins, which may contribute to its higher bioavailability compared to Fe (69). The differential distribution and chemical speciation of these minerals account for the relatively higher bioavailability of Zn, estimated at approximately 25%, compared to Fe, which stands at approximately 10% (70). The prominence of minerals in the aleurone layer and embryo, coupled with their limited bioavailability, poses a significant constraint on improving the nutritional contribution of wheat through conventional genetic approaches. This challenge has led to the exploration of genetic engineering as a viable solution to enhance mineral bioavailability.

A noteworthy study conducted by Wan et al. (71) exemplified the potential of genetic engineering in enhancing the nutritional quality of wheat. In this study, a genetically enhanced wheat line was developed, resulting in elevated levels of Zn and Fe in its grains. Importantly, the distribution patterns of these minerals in the biofortified wheat line were comparable to those in control lines. Zn was notably concentrated in the embryo and axis of the grain, aligning with its higher bioavailability. Additionally, iron and Zn contents exhibited correlations with phosphorus levels, indicating a potential relationship between these minerals and phosphorus that could influence their bioavailability. This suggests that the bioavailability of these minerals is unlikely to significantly differ between the biofortified and control wheat lines, providing optimism for the use of biofortified wheat as a potential nutritional solution. In parallel, a transgenic approach was pursued, incorporating the TaVIT2-D wheat gene and the OsNAS2 rice gene. This approach led to a significant increase in Zn concentration and an alteration in iron distribution within white-flour fractions. The VIT-NAS construct effectively doubled Zn content in wholemeal flour to approximately 50 μg g − 1. Although the total iron content remained unchanged, there was a three-fold increase in highly pure, roller-milled white flour, reaching around 25 μg g − 1. Notably, OsNAS2 expression partially restored iron distribution to the aleurone layer, which is traditionally iron-depleted in grains overexpressing TaVIT2 alone. This transgenic approach also resulted in elevated levels of nicotianamine in VIT-NAS grains, further enhancing iron and Zn bioaccessibility in white flour. The growth of VIT-NAS plants closely mirrored that of untransformed controls, reinforcing the potential for enhancing wheat’s nutritional quality through genetic engineering (72).

Taken together, the spatial distribution and chemical speciation of minerals, particularly iron and zinc, within wheat grains play a pivotal role in determining their bioavailability. While conventional genetic improvement faces substantial challenges due to these limitations, genetic engineering offers a novel avenue to concentrate these vital minerals in the starchy endosperm, thereby enhancing their bioavailability and potentially addressing widespread micronutrient deficiencies. This innovative approach holds great promise for improving the nutritional quality of wheat and, consequently, human health.

## 5 Progress in assessing the bioavailability of Fe and Zn: advancements in *in vitro* and *in vivo* methodologies

The primary objective of wheat Fe and Zn biofortification programs is to enhance the nutritional quality of wheat grains by increasing their Fe and Zn content, with a specific focus on addressing malnutrition among vulnerable populations, particularly children and pregnant women. However, the efficacy of these programs hinges on the bioavailability of Fe and Zn in the enriched grains, as these nutrients must be readily absorbed by the human intestine to be nutritionally beneficial. To achieve this, it is crucial to establish and utilize robust methodologies for assessing Fe and Zn bioavailability, which constitutes a fundamental aspect of wheat biofortification initiatives. Two principal approaches for evaluating Fe and Zn bioavailability and bioaccessibility are *in vivo* and *in vitro* methods. The *in vitro* approach, specifically estimating bioaccessibility (the portion of nutrients released from the food matrix and available for absorption), has gained significant attraction. This method involves simulating the human gastrointestinal digestion process, encompassing both gastric and intestinal digestion phases (73). The *in vitro* approach holds particular significance as it enables plant breeders to identify promising materials within large wheat breeding populations at an early stage of generation advancement. Compared to the *in vivo* approach, the *in vitro* method offers advantages in terms of speed, cost-effectiveness, and the ability to screen a substantial pool of wheat genotypes within a defined timeframe.

The strong positive correlation observed between *in vivo* and *in vitro* approaches underscores the potential for large-scale *in vitro* screening of wheat genotypes to expedite targeted breeding efforts. The assessment of *in vitro* bio-accessibility, particularly the solubility and dialyzability of Fe (74) and Zn (75), is the favored method due to its substantial positive correlation with *in vivo* results regarding human absorption (Fe: *r* = 0.89, Zn: *r* = 0.925). Over time, studies have been dedicated to refining *in vitro* methodologies for assessing Fe and Zn bio-accessibility in food crops (Supplementary Table S5). Ferruzzi et al. (76) recently provided a comprehensive review of commonly employed *in vitro* approaches for measuring MNs bioavailability in cereals and related foods. In a commendable effort, Brodkorb et al. (77) sought to harmonize *in vitro* digestion protocols to enhance comparability of food digestibility and MNs bioaccessibility assessments across laboratories. This harmonization involved standardizing reagent selection and sourcing, as well as establishing consistent conditions for gastric and small intestinal phases, potentially serving as a useful model to address inter-laboratory variations. In the realm of bioavailability studies, iron bioavailability has been extensively investigated using both *in vitro* and *in vivo* models, including human studies (78). However, it is noteworthy that despite the existing body of research, there has been limited progress in enhancing the methodologies for rapid and precise estimation of Fe and Zn bioavailability in wheat.

Prior to 2015, the predominant focus in research was on examining the solubility, dialyzability, and bioaccessibility of Fe and Zn in fortified wheat-based end-products, as opposed to biofortified grain flour. This disparity can be attributed to the limited availability of biofortified wheat varieties (Table 2). Even from 2015 to 2022, despite the introduction of several biofortified wheat varieties, there has been limited advancement in this area, as evidenced by existing research data. For instance, Rebellato et al. (79) demonstrated varying levels of Fe solubility (ranging from 0.12 to 0.43 mg kg^−1^) and dialyzability (ranging from 0.02 to 0.21 mg kg^−1^) in 41 different fortified biscuits. Rodriguez-Ramiro et al. (80) reported an eightfold increase in Fe release from bread produced using a sourdough process compared to conventional yeast and Chorleywood Bread-Making Process, as determined through *in vitro* dialyzability and a Caco-2 cell model. Additionally, Lu et al. (81) employed a trivariate model based on Zn homeostasis within the human intestine to reveal substantial variations in Zn bioaccessibility among 30 wheat lines cultivated at two different locations.

**Figure 5:**
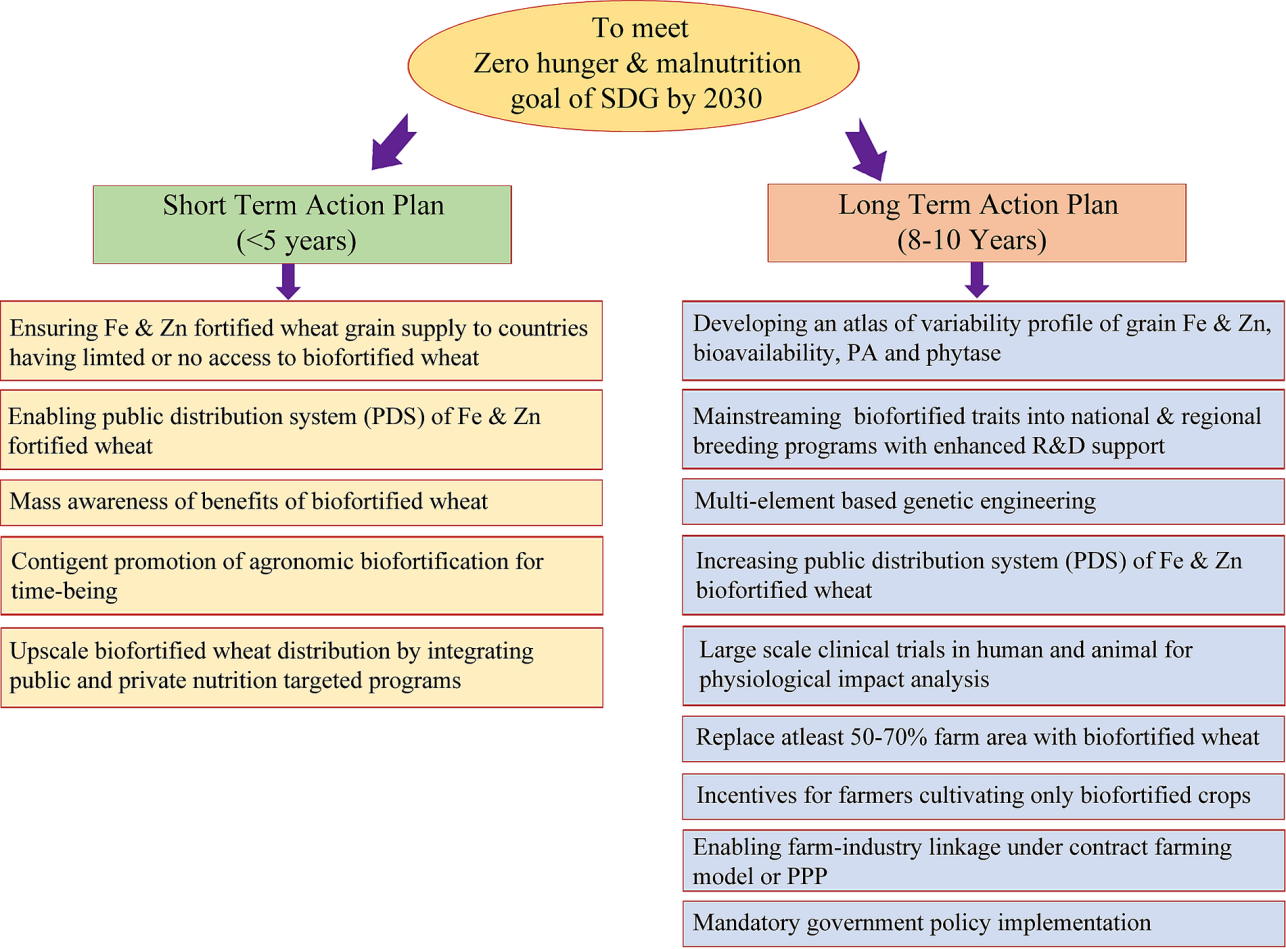
Proposed short-term and long-term action plant/ working model to meet the zero hunger and malnutrition target of SDG by 2030.

Surprisingly, our search yielded no reports pertaining to comprehensive large-scale bioavailability assessments of Fe and Zn biofortified wheat varieties. Consequently, as emphasized in Figures 4, 5, the need for extensive Fe and Zn bioavailability assays has been underscored, indicating a promising avenue for future research. This endeavor involves the development of optimized and efficient *in vitro* methodologies, which can effectively complement existing *in vivo* approaches. The potential benefits of these *in vitro* methods are significant. They could expedite the selection of wheat genotypes with superior Fe and Zn bioavailability within extensive breeding populations, thereby reducing the burden on early breeding generations. It is recommended that wheat breeders routinely incorporate bioavailability assessments, including those of biofortified varieties, utilizing established *in vitro* techniques. This integration has the potential to augment breeding efficiency in the creation of wheat cultivars enriched with Fe and Zn and exhibiting enhanced bioavailability. Furthermore, collaborative efforts between plant breeders, food scientists, and nutritionists are imperative. These collaborations should focus on both quantifying Fe and Zn content in wheat grain and elucidating their bioavailability. Such endeavors aim to establish consensus regarding appropriate target levels for Fe and Zn in wheat varieties, providing crucial guidance for breeders in their pursuit of optimizing nutrient content and bioavailability.

## 6 Impact analysis of Fe and Zn bio-fortified wheat on human and animal health

The utilization of human and animal model-based methodologies stands as the most suitable approach, when compared to alternative methods, for the assessment of the impact of Fe and Zn bio-fortified food products. In this context, it is hypothesized that trials conducted in both animal and human subjects have yielded positive outcomes regarding the enhancement of MN levels. Accordingly, this section aims to elucidate the potential intricacies associated with the utilization of distinct animal and human model systems for evaluating the effects of administering Fe and Zn bio-fortified wheat. It also delves into the advantages and disadvantages inherent in each approach. Furthermore, the section provides insights into clinical trials conducted in humans, with a specific focus on the effects observed in participants following the consumption of wheat bio-fortified food products.

### 6.1 Proof-of-concept from animal trials

Animal models are valuable tools for studying the *in vivo* bioavailability of essential minerals like Fe and Zn and for gaining insights into gene expression changes induced by the consumption of biofortified foods. Several animal models, including rats (*Rattus norvegicus*), poultry (*Gallus gallus*), and piglets (*Sus scrofa*), have been employed to assess the bioavailability of micronutrients (MNs) in various food matrices (82, 83). Rats are useful for comparative evaluations of bioavailable Fe and Zn in plant-based foods. However, it’s important to note that rats tend to overestimate bioavailability when compared to humans due to their ability to synthesize vitamin C (which enhances Fe absorption) and phytase (which further enhances Fe absorption) (82). In contrast, poultry is frequently employed in *in vivo* bioavailability studies because they exhibit rapid physiological and clinical responses to low Fe levels (83). Piglets, owing to their physiological and anatomical similarity to humans in terms of gastrointestinal features, are also considered excellent models. Considering the strengths and limitations of each animal model, our hypothesis suggests that poultry and piglets may offer greater potential as *in vivo* models for investigating the physiological variations induced by biofortified foods.

Over the past decade, limited research has focused on assessing the physiological and clinical effects of Fe and Zn bio-fortified wheat using animal models due to the scarcity of such bio-fortified wheat varieties. Consequently, prior to 2015, most studies primarily investigated the bioavailability of Fe and Zn in fortified wheat products. For example, Welch et al. (84) employed *Rattus norvegicus* (Norwegian brown rat) to assess the absorption and retention of Zn from 28 wheat genotypes with varying Zn content (ranging from 33 to 149 mg kg-1) and Fe content (ranging from 80 to 368 mg kg-1). Their results revealed significant differences in the bioavailability of 65Zn, ranging from 60 to 80%, with genotypes containing higher Zn levels exhibiting greater bioavailability compared to low Zn genotypes. Similarly, Carlson et al. (85) observed substantial variation in the solubility of Fe (ranging from 3 to 11%) and Zn (ranging from 34 to 63%) in pigs fed with three different wheat types (control, low Zn, and high Zn), suggesting a positive correlation between Zn bioavailability and the Zn content in wheat grains. While these animal model-based Fe and Zn bioavailability studies provide promising but limited insights, drawing definitive conclusions and recommendations remains biased. Further research is imperative, particularly investigating the physiological and clinical impacts of bio-fortified wheat varieties. Moreover, recent reports have highlighted the influence of Zn- and Fe-enriched foods on the composition of gut, intestinal, and cecal microbiota in various animal models, subsequently modulating the host’s Zn status (86). Nevertheless, investigations regarding the impact of Fe and Zn bio-fortified wheat on microbiota composition in animal models are scant. For example, Reed et al. (87) demonstrated an increase in β-microbial diversity, particularly with members of *Dorea*, *Clostridiales*, *Ruminococcus*, *Lactobacillus reuteri*, and *Lachnospiraceae*, in the cecum of *Gallus gallus* (chickens) following a six-week efficacy trial involving Zn-bio-fortified wheat (46.5 μg Zn/g). Similarly, Beasley et al. (88) reported an enhancement in the composition of *Actinobacteria* and a decrease in pathogenic bacteria such as *Proteobacteria, Firmicutes, Escherichia*, and *Streptococcus* in *Gallus gallus* fed with Fe bio-fortified wheat during a six-week efficacy trial. However, additional research in this area is warranted to comprehensively understand the effects of Fe and Zn bio-fortified wheat on microbiota composition in animal models.

Since Zn is necessary to sustain the metabolic growth in bacteria, a large magnitude of Zn-dependent microbes is scaled up in Zn abundant atmosphere. This could potentially have favorable consequences on the host’s gut and intestinal microbial makeup, with no undesirable reactions on the genetic capacity. Moreover, gut microbiome linked with Zn-bio-fortified wheat consumption is exclusive and might change host Zn composition, reduce pathogenic bacteria and consequently improve gut health. Based on preliminary reports on the impact of Zn-bio-fortified wheat on gut microbiome and host Zn status, a large scale impact analysis in different animal models involving Fe-bio-fortified and Zn-bio-fortified wheat independently and in combination would provide sizable data to perform statistical meta-analysis to draw meaningful inferences on physiological, microbial and clinical outcomes. This might help us devise a possibly more efficient approach to combat MNs deficiencies and improve overall health.

### 6.2 Proof-of-concept from human trials

Randomized controlled trials (RCTs) are a preferred method for evaluating the nutritional effects of biofortified wheat at the consumer level, distinguishing them from conventional assessment approaches. Table 3 provides an overview of RCTs focused on assessing the impact of biofortified wheat. These clinical trials, following the RCT philosophy, are a popular yet resource-intensive and time-consuming means to measure changes in participants’ characteristics, such as height, weight, cognitive parameters, and immune system function, in comparison to non-biofortified counterparts. Typically, RCTs involve a vulnerable group, particularly children and lactating women, who are the target beneficiaries of nutritional programs. However, non-lactating women have also participated in similar studies (90). In assessing nutrients like Vitamin-A, visual adaptation to darkness is employed, whereas for Fe and Zn, participants’ physical activities and cognitive changes are observed. RCTs in this context are intricate, and their outcomes become apparent only after a substantial duration (93). In these human trials, participants in the test group are required to adhere to a specific diet compared to a control group, often referred to as ‘placebo.’ This comparison between the ‘test’ and ‘placebo’ groups helps confirm the nutritional impact of biofortification. Recognizing its significant importance, there has been a recent upsurge in the conduct and registration of RCTs through platforms like ClinicalTrials.gov, reflecting the growing emphasis on MN research. This section provides an account of the current status of clinical studies conducted in humans related to biofortified wheat, acknowledging their limited number but substantial relevance in assessing the nutritional benefits of such crops.

**Table 3:** An overview of RCTs on analyzing the impact of bio-fortified wheat.

Country	RCT period	Sample size	Nutrient	Form of treatment	Reference
Mexico	Short-term (2 days)	27	Zn	Wheat Tortillas (Test Meal)	Rosado et al. (89)
India	9 months	284	Zn	Wheat Flour (Zn Foliar Application)	NCT02241330 (2016)
India	6 months	6000	Zn	Wheat Flour (PBW 550)	Sazawal et al. (90)
Switzerland	13 months	73	Zn	Whole Wheat Porridge (Test Meal)	Signorell et al. (91) NCT01775319
Pakistan	12 months	482	Zn and Fe	Wheat Flour	Lowe et al. (92)
Ghana	11 months	1073	Zn and Fe	Bouillon Cubes (with wheat flour)	NCT04632771 (2021)

Rosado et al. (89) conducted a study on Mexican adult women to assess the increase in absorbed Zn levels resulting from the consumption of Zn-rich biofortified wheat meals for two consecutive days (breakfast, lunch, and dinner). They also examined the contribution of dietary Zn and PA to Zn absorption rates. The findings revealed a significant 72% increase (5.7 mg/day) in Zn intake at a 95% extraction rate and a 68% increase (2.7 mg/day) at an 80% extraction rate compared to the control group. Zn absorption rates were estimated at 2.1 ± 0.7 mg/day and 2.0 ± 0.4 mg/day for the 95 and 80% extraction rates, respectively. In both cases, the test group showed a 0.5 mg/day increase in Zn absorption compared to the control group, demonstrating the positive impact of biofortified wheat. In 2016, a study targeting Indian schoolchildren aged 6–12 with low Zn levels (NCT02241330, 2016^5^) assessed the efficacy of Zn biofortified wheat combined with fortified food compared to a control group of 284 participants. The study aimed to measure changes in plasma Zn levels from baseline to the intervention endpoint and 2 months post-intervention. Sazawal et al. (90) conducted a RCT involving 6,000 participants, including healthy non-pregnant, non-lactating women aged 15–49 and preschool children aged 4–6, to evaluate the efficacy of Zn-enriched biofortified wheat over 6 months. The study revealed that children consuming biofortified wheat experienced fewer illness days, with a 17% reduction in pneumonia-related illness days and a 39% decrease in vomiting days compared to the control group. Among women respondents, there was a 9% reduction in fever-related illness days. Signorell et al. (91) conducted a study involving 73 participants from Switzerland, including 18 men and women and 55 women aged 18–45. Their research showed that Zn absorption from biofortified wheat, achieved through agronomic intervention, yielded similar results to fortified wheat. The study compared Zn absorption between the biofortified wheat and fortified control groups and found minimal differences in Zn absorption between intrinsic and extrinsic labels.

Further, these studies conclude that, irrespective of intervention, there is a substantially significant Zn absorption (higher by 70–76%) in humans, with no effect on extraction rate. A majority of the clinical trials to test the efficacy of bio-fortification have been conducted in Asian and African countries due to the amount of undernourished population compared to the other regions. In one of the ongoing studies in Pakistan, Zn & Fe status is proposed to be measured as an outcome of consuming bio-fortified wheat flour by 509 adolescent/child pairs selected from 482 households (92). The study’s primary goal was to upscale bio-fortified wheat by estimating the status of Zn via bio-markers (functional and biochemical) and assessing Fe′s status as a secondary outcome. An efficacy study was started in Ghana on Oct 19, 2020 (NCT04632771, 2021^6^) to assess the nutritional status of 1073 participants comprising women of 15–49 years of age (non-pregnant, non-lactating of reproductive age and lactating) and preschool children of 2–5 years of age is in progress. In the ongoing study, intervention is through feeding the multiple micronutrients fortified bouillon cube with wheat flour as one of the ingredients.

Literature on the aforementioned animal and human trials concerning wheat bio-fortification is minimal. Even in the available literature, information like baseline data, standard error, and standard deviation are missing in one or another, thereby restricting performing meta-analysis to provide a statistical evidence-based concrete conclusion. Yet, the outcome of individual trials in animals or humans shows the positive effect of wheat bio-fortification, leading to acceptance of the proposed hypothesis. Overall, such efficacy studies provide evidence to prescribe policies targeted for regional, national, and international development.

## 7 Policy framework for scaling-up bio-fortification in wheat

To combat MNDs, biofortification stands as a cost-effective and widely endorsed solution. Expanding biofortification efforts in wheat, a key dietary staple, necessitates a robust policy framework for coordinated implementation among stakeholders (94). Firstly, it is imperative to bolster research and development (R&D) organizations engaged in wheat biofortification through increased investment. These efforts should seamlessly integrate with breeding programs, either at the regional or national level (94, 95). Concurrently, rigorous, evidence-based impact assessments must be conducted to elucidate the potential benefits of biofortification. Secondly, global regulatory authorities should harmonize frameworks, particularly in the context of clinical trial protocols spanning multiple countries. Thirdly, raising awareness among stakeholders is essential. Integration of biofortified wheat into conventional regional and national food supply chains, facilitated by providing biofortified wheat seeds to farmers, can promote widespread adoption. Offering premium prices or financial incentives for biofortified crop yields can further stimulate uptake (93). Lastly, on the consumption front, aggressive promotional initiatives, including nutrient-profile-based food labeling and nutrition awareness campaigns, should be employed. Additionally, incorporating biofortified “combo packs” into national nutrition or safety net programs, such as combinations of wheat and legumes, can unlock the potential benefits of biofortification (96). To promote the consumption of bio-fortified wheat, strategic alliances can be established with food industry stakeholders, and media campaigns can be initiated to drive consumer demand. In countries like India, where public procurement is prevalent, it is essential to introduce a system of segregated procurement of bio-fortified grains by authorized agencies or segregated trading by private millers to ensure consumers have access to these nutritious grains. Local and national governments should play a facilitating role in promoting access to bio-fortified wheat, particularly in regions grappling with persistent undernutrition and food shortages, by implementing trade promotion initiatives.

## 8 Conclusions and way forward

Recognizing the nutritional importance of wheat and the significant variability in Fe and Zn content within the global wheat supply, extensive efforts spanning agronomic, genetic and conventional breeding, molecular and transgenic techniques, as well as microbial interventions, have yielded substantial insights into wheat biofortification. These efforts have culminated in the development and commercial cultivation of 40 biofortified wheat cultivars in India, Pakistan, Bangladesh, and Mexico, aimed at mitigating MND. Figure 5 outlines a comprehensive short-term and long-term action plan to align with the Sustainable Development Goal of achieving zero hunger and alleviating malnutrition by 2030, capitalizing on the nutritional potential of wheat. Over the past decade, the advent of high-throughput omics technologies has facilitated the decoding of the intricate wheat genome, unveiling a plethora of uncharacterized genes and omics-based information. However, the full utilization of this genetic wealth in wheat Fe and Zn biofortification breeding programs remains an ongoing endeavor. Biofortification stands out as a sustainable and cost-effective strategy for addressing MND, particularly in economically disadvantaged populations, in comparison to traditional fortification methods. Table 4 provides an overview of initiatives in wheat biofortification and fortification over the past 5 years. While promising results from limited-scale human and animal trials suggest the positive impact of biofortified wheat cultivars, broader validation on a larger scale with harmonized protocols is imperative for conclusive evidence and wider adoption.

**Table 4:** List of initiatives during the last 5 years (2017–2022) on wheat bio-fortification and fortification.

Initiatives	Objectives	Status
Production and dissemination of bio-fortified crop varieties	To produce high-quality seed and ensure availability of quality planting to farmers	290 varieties of 12 different bio-fortified crops, including wheat (approx. 40 varieties; refer Table 2) released in 30 countries and tested in more than 60 countries Reaching nearly 10 million farm households and providing more than 50 million farming households with access to bio-fortified foods
Standardization and regulation	To integrate (bio)fortification into global standards and guidelines	Like Codex Alimentarius’ food standards, efforts are underway to integrate bio-fortification into global standards and guidelines The Food Safety and Standard Authority of India (FSSAI) has introduced the ^+^F logo for fortified staple food products and formulated a defined set of standards for fortification Partners: Codex Alimentarius, food standards-setting agency, WHO, FAO, World Trade Organization etc.
Integrated approach	Involvement of multi-stakeholder platforms	The World Bank encourages nutrition-sensitive agricultural initiatives, including bio-fortification, e.g., Global Donor Platform for Rural Development ‘Purchase for Progress’ by the World Food Program (WFP). In Rwanda, local iron bean production is purchased and stored in WFP warehouses for later emergencies The WHO Nutrition Guidance Expert Advisory Group issued guidelines on bio-fortification as a public health nutrition intervention The African Development Bank’s new “Banking on Nutrition” technical partnership
Private sector and NGO	Robust private seed systems and NGOs in linkage to HarvestPlus	HarvestPlus has MOUs with private seed companies and NGOs Private sector seed companies help in marketing, developing and testing bio-fortified varieties E.g. The food value chain Nigeria cassava is headed by small and medium-size food processors World Vision (NGO), along with Harvestplus, is working in 15 countries
Government involvement	Policy level implementation by Government entities for integration into their agriculture and nutrition policies	HarvestPlus is working closely with government-sponsored bio-fortification programs in Brazil, China, and India HarvestPlus, in collaboration with Latin American and Caribbean (LAC) program, provides technical assistance and support to government-driven bio-fortification programs in Bolivia, Colombia, Guatemala, Haiti, Nicaragua, and Panama and is exploring efforts in other target countries.
Awareness campaign *cum* nutritional messaging	Filed days, demonstrations, mass media campaigns to men and women; seed village’ model and strengthening the linkages with agrifood-processing industry	In India, Nutrition International (NI) is providing technical support for fortification Food fortification Resource Center (FFRC) provides technical support concerning technology, premix, equipment procurement and creates awareness among consumers on good nutrition, food safety and fortification.
Fortification	To fortify wheat flour produced in industrial mills with vitamins and minerals	>80 countries have legislated rules and regulations related to wheat flour fortification The government of India has mandated the use of fortified wheat flour into its Integrated Child Development Scheme (ICDS) and Mid-Day Meal Scheme (MDM), and Public Distribution System (PDS) 250 million beneficiaries, including pregnant and lactating mothers and children up to the age of 13, can be reached directly

Source (97–100); HarvestPlus (http://www.harvestplus.org/), https://blog.nextias.com/food-fortification-in-india (accessed on 13 Jan, 2022).

Despite this success, the challenges remain the same, i.e., increasing wheat grain Fe and Zn content and their bioavailability. Therefore, future efforts can be based on two key strategies, i.e., 1. developing Fe and Zn rich wheat genotypes with improved bioavailability by utilizing the available physiological, biochemical, and molecular knowledge in wheat and other related species; and 2. generating new knowledge on Fe and Zn homeostasis in wheat starting from soil to grain, by utilizing the available wheat genome and advanced omic-based technologies. As the first strategy could ensure the timely availability of Fe and Zn enriched wheat cultivars to various stakeholders, strategy two will strive to generate new knowledge that wheat breeders could utilize to develop even superior wheat lines with high grain Fe and Zn having greater bioavailability. Both the strategies are supplementary and cyclic in nature, which could efficiently maintain the anticipated pace of the whole bio-fortification program to address global MND in a stipulated time frame. Generating new knowledge may include areas such as functional characterization of genes using emerging technologies such as CRISPR/Cas system, characterizing wild relatives and germplasms as a part of pre-breeding, creating new genetic variations using mutation breeding, developing faster and accurate *in vitro* methodologies to estimate Fe and Zn content and bioavailability in early breeding generations, minimizing the harmful effect of anti-nutritional factors from grain and end-products, evaluating the physicochemical, rheological and baking properties of bio-fortified end-products, the health impact analysis of bio-fortified wheat by conducting large-scale human and animal trials. By pursuing these avenues of research and innovation, we can accelerate progress toward addressing global MNDs within the specified time frame.

## Author contributions

OG: Conceptualization, Data curation, Formal analysis, Investigation, Methodology, Writing – original draft, Writing – review & editing. AS: Data curation, Formal analysis, Methodology, Writing – original draft. VP: Data curation, Methodology, Validation, Writing – original draft. RS: Data curation, Formal analysis, Methodology, Writing – original draft. MK: Data curation, Investigation, Methodology, Writing – original draft. AP: Data curation, Methodology, Writing – review & editing. SK: Formal analysis, Writing – review & editing. MH: Writing – review & editing. SR: Project administration, Supervision, Validation, Writing – review & editing. GS: Funding acquisition, Investigation, Project administration, Resources, Supervision, Writing – review & editing.

## Funding

The author(s) declare financial support was received for the research, authorship, and/or publication of this article. Authors are grateful to the Indian Council of Agricultural Research, Department of Agricultural Research and Education, Government of India for providing financial help under grant no. 1006422, institutional project (CRSCIIWBRSIL 201500900190) and Department of Biotechnology, Government of India under the grant BT/NABI-Flagship/2018 and No. BT/Ag/Network/Wheat/2019-20.

## Footnotes

^1^https://apps.fas.usda.gov/psdonline/circulars/grain.pdf (Retrieved 17/09/2023).

^2^http://www.fao.org/faostat/en/#data (Retrieved 28/11/ 2022).

^3^http://www.ars.usda.gov/nea/bhnrc/mafcl

^4^http://www.fortificationdata.org. (Retrieved 18/02/2022).

^5^https://clinicaltrials.gov/ct2/show/NCT02241330

^6^https://clinicaltrials.gov/ct2/show/NCT04632771
