# Supplementary material for: Critical assessment of wheat biofortification for iron and zinc: a comprehensive review of conceptualization, trends, approaches, bioavailability, health impact, and policy framework
Source: Front Nutr. 2024 Jan 4;10:1310020. doi: 10.3389/fnut.2023.1310020 (PMC10794668; doi:10.3389/fnut.2023.1310020)
Supplement: Supplementary file 1 [file Table_1.DOCX]

**Table S1:** RDA of Fe, Zn and folate in children and women (adopted from Institute of Medicine, 1998; 2001 and NIH, 2020).

| **Age group** | **RDA Fe (mg)** | | **RDA Zn (mg)** | | **RDA Folate (µg DFE*)** | |
| --- | --- | --- | --- | --- | --- | --- |
|  | **Male** | **Female** | **Male** | **Female** | **Male** | **Female** |
| Birth to 6 months | 0.27 | | 2 | | 65 | |
| 7-12 months | 11 | | 3 | | 80 | |
| 1-3 years | 7 | | 3 | | 150 | |
| 4-8 years | 10 | | 5 | | 200 | |
| 9-13 years | 8 | | 8 | | 300 | |
| 14-18 years | 11 | 15 | 11 | 9 | 400 | |
| 19^+^ years | 8 | 18 | 11 | 8 | 400 | |
| Pregnancy | - | 27 | - | 12 | 600 | |
| Lactation |  | 10 |  | 13 | 500 | |

*1 mcg DFE = 0.6 mcg folic acid from fortified foods or dietary supplements consumed with foods
