# Supplementary material for: Critical assessment of wheat biofortification for iron and zinc: a comprehensive review of conceptualization, trends, approaches, bioavailability, health impact, and policy framework
Source: Front Nutr. 2024 Jan 4;10:1310020. doi: 10.3389/fnut.2023.1310020 (PMC10794668; doi:10.3389/fnut.2023.1310020)
Supplement: Supplementary file 2 [file Table_2.DOCX]

| **Region** | **% Stunting (number in million)** | **% Wasting (number in million)** | **% Overweight (number in million)** |
| --- | --- | --- | --- |
| **Africa** | **29.1 (57.5)** | **1.8 (3.5)** | **4.7 (9.3)** |
| Eastern Africa | 34.5 (23.1) | 1.1 (0.7) | 3.7 (2.5) |
| Middle Africa | 31.5 (9.5) | 2.2 (0.7) | 5.1 (1.5) |
| Northern Africa | 17.6 (5.1) | 3.1 (0.9) | 11.3 (3.3) |
| Southern Africa | 29.0 (2.0) | 0.9 (0.1) | 12.7 (0.9) |
| Western Africa | 27.7 (17.8) | 1.8 (1.1) | 1.9 (1.2) |
| **Asia** | **21.8 (78.2)** | **2.9 (10.5)** | **4.8 (17.2)** |
| Central Asia | 9.9 (0.8) | 0.6 (0.1) | 6.2 (0.5) |
| Eastern Asia2 | 4.5 (4.1) | 0.4 (0.4) | 6.3 (5.7) |
| Southern Asia | 31.7 (55.9) | 4.4 (7.8) | 2.5 (4.5) |
| South-eastern Asia | 24.7 (13.9) | 3.6 (2.0) | 7.5 (4.2) |
| Western Asia | 12.7 (3.4) | 1.1 (0.3) | 8.4 (2.3) |
| **Latin American and Caribbean** | **9.0 (4.7)** | **0.3 (0.1)** | **7.5 (3.9)** |
| Caribbean | 8.1 (0.3) | 0.9 (0.0) | 7.0 (0.2) |
| Central America | 12.6 (2.0) | 0.2 (0.0) | 6.9 (1.1) |
| South America | 7.3 (2.4) | 1.3 (0.4) | 0.2 (0.1) |
| **Global** | **21.3 (144.0)** | **2.1 (14.3)** | **5.6 (38.3)** |

**Table S2**: Global prevalence of stunting, wasting and overweight under 5 year’s children.

**$ as per the estimate of 2019 (Source:** UNICEF-WHO-The World Bank, 2020).
