# Supplementary material for: Critical assessment of wheat biofortification for iron and zinc: a comprehensive review of conceptualization, trends, approaches, bioavailability, health impact, and policy framework
Source: Front Nutr. 2024 Jan 4;10:1310020. doi: 10.3389/fnut.2023.1310020 (PMC10794668; doi:10.3389/fnut.2023.1310020)
Supplement: Supplementary file 3 [file Table_3.DOCX]

**Table S3:** Chronological progress and trends of wheat flour fortification with Fe, Zn and folate.

| **Micronutrients** | | **Sources of fortificants** | **Results** | **References** |
| --- | --- | --- | --- | --- |
| Fe | | Meal was prepared using a combination of four green leafy vegetables (amaranthus, cabbage, spinach and fenugreek) with cereals (wheat, rice, sorghum, maize, pearl millet and finger millet), one at a time | Average bioavailable Fe density of green leafy vegetables based meals was significantly higher | Chiplonkar et al. (1999) |
| Fe | | 66 mg/kg of electrolytic or reduced Fe | Fortification of flour with electrolytic Fe or reduced Fe was not beneficial in reducing anaemia in preschoolers due to the low prevalence of anaemia and low bioavailability of the fortificant Fe | Nestel et al. (2004) |
| Folic acid | | 0.2 mg folic acid/100 g product | Baking processes from flour to bread reduced folic acid in a range from 12 to 21% | Gujska and Majewska (2005) |
| Fe | | - | Fortification had no effect on haemoglobin levels of the children studied since baseline mean haemoglobin was 11.3±2.8 g/dL, and post-fortification mean haemoglobin was 11.2±2.8 (at 12 months) and 11.3±2.5 g/dL (at 24 months). | Assunção et al. (2007) |
| Fe and Zn | | NaFeEDTA, elemental Fe, ZnSO_4_ and ZnO | Fortification significantly decreased moisture and protein content and increased ash content. Fortificants exerted a slight deteriorative effect on texture characteristics of the chapatis | Akhtar et al. (2008) |
| Fe | | FeSO_4_ | In the proportion of 3:1 fortified food: meat examined, meat increases the bioavailability of Fe-fortified foods | Pachón, Stoltzfus, and Glahn (2008) |
| Fe | | - | Mid-term evaluation in Iran indicated that the Fe fortification programme has only had a beneficial effect on the prevalence of low ferritin levels. | Sadighi et al. (2008) |
| Zn and Se (selenium) | | A hull-less barley flour and flakes were incorporated into white and whole-grain wheat bread | The supplemented bread was high in fibre, Zn and Se content | Škrbić et al. (2009) |
| Folic acid | | 1mg/kg wheat flour | Significantly reduced the prevalence of folate deficiency in Australia | Brown et al. (2011) |
| Folic acid | | 1.8mg/kg of wheat flour | Effectively reduced neural tube defect (NTDs) | Cortes, Mellado, and Pardo (2012) |
| Fe | | Small rolls of 20g with 4mg basic Fe content per unit as microencapsulated FeSO_4_ | Well tolerated, increased haemoglobin levels and reduced the occurrence of anaemia | Barbosa et al. (2012) |
| Fe | | Mean value of 5.4mg/100 g of wheat flour from different manufactures | Some manufacturers do not perform adequate control of Fe enrichment approach | Buzzo et al. (2012) |
| Fe, Zn and Ca | | FeSO_4,_ ZnSO_4_ and CaCO_3_ @ 40, 20 and 1000 mg/kg of wheat flour | The Fe and Zn levels were significantly higher in rats' plasma, liver, and femur fed with Fe and Zn fortified bread. Interaction of Ca, Fe and Zn resulted in their decreased bioavailability. | [Ahmed](https://www.ncbi.nlm.nih.gov/pubmed/?term=Ahmed%20A%5BAuthor%5D&cauthor=true&cauthor_uid=24293693), et al. (2012) |
| Folic acid | 3mg/kg of wheat flour | | Significant improvements in folate status among women of reproductive age | Noor et al. (2017) |
| Fe | FeH_2_O_5_S, C_4_H_2_FeO_4_, Fe^2+^, NaFeEDTA, microencapsulated FeSO_4_, and microencapsulated C_4_H_2_FeO_4_ | | NaFeEDTA proved to be the most effective iron compound in the fortification of wheat flour | Rebellato et al. (2017) |
|  |  |  | Ferrous sulfate and microencapsulated compounds affected flour quality parameters, while reduced iron was the compound that promoted the most stability to flour. | Rebellato et al. (2018) |
| Fe | Microencapsulated FeSO_4_ with or without ascorbate | | The bioaccessibility of the iron after digestion of the fortified bread varied from 41.45 to 99.31% | Bryszewska et al. (2019) |
| Fe | Three trials used 41 mg to 60 mg Fe/kg flour, three trials used <40 mg Fe/kg and three trials used >60 mg Fe/kg flour. | | Fe‐fortified wheat flour with or without other micronutrients added versus wheat flour (no added Fe) with the same other micronutrients added may reduce anaemia risk by 27% in populations | Field et al. (2020) |

**Reference**

1. [Ahmed](https://www.ncbi.nlm.nih.gov/pubmed/?term=Ahmed%20A%5BAuthor%5D&cauthor=true&cauthor_uid=24293693), A., F. M. [Anjum](https://www.ncbi.nlm.nih.gov/pubmed/?term=Anjum%20FM%5BAuthor%5D&cauthor=true&cauthor_uid=24293693), M. A. [Randhawa](https://www.ncbi.nlm.nih.gov/pubmed/?term=Randhawa%20MA%5BAuthor%5D&cauthor=true&cauthor_uid=24293693), U. [Farooq](https://www.ncbi.nlm.nih.gov/pubmed/?term=Farooq%20U%5BAuthor%5D&cauthor=true&cauthor_uid=24293693), S. [Akhtar](https://www.ncbi.nlm.nih.gov/pubmed/?term=Akhtar%20S%5BAuthor%5D&cauthor=true&cauthor_uid=24293693), and M. T. [Sultan](https://www.ncbi.nlm.nih.gov/pubmed/?term=Sultan%20MT%5BAuthor%5D&cauthor=true&cauthor_uid=24293693). 2012. Effect of multiple fortification on the bioavailability of minerals in wheat meal bread. *Journal of Food Science and Technology* 49 (6): 737–744.
2. Akhtar, S., F. M. Anjum, S. U. Rehman, M. A. Sheikh, and K. Farzana. 2008. Effect of fortification on physic chemical and microbiological stability of whole wheat flour. *Food Chemistry* 110: 113-119.
3. Assuncao, M. C., I. S. Santos, A. J. Barros, D. P. Gigante, and C. G. Victora. 2007. Effect of iron fortification of flour on anemia in preschool children in Pelotas, Brazil. Revista de Saúde Pública 41 (4): 539-48.
4. Barbosa, T. N. N., J. A. A. C. Taddei, D. Palma, F. Ancona-Lopez, and J. A. P. Braga. 2012. Double-blind randomized controlled trial of rolls fortified with microencapsulated iron. *Revista da Associação Médica Brasileira* 58: 118-124.
5. Brown, R. D., M. R. Langshaw, E. J. Uhr, J. N. [Gibson](https://pubmed.ncbi.nlm.nih.gov/?term=Gibson+JN&cauthor_id=21241218), and [D. E. Joshua](https://pubmed.ncbi.nlm.nih.gov/?term=Joshua+DE&cauthor_id=21241218). 2011. The impact of mandatory fortification of flour with folic acid on the blood folate levels of an Australian population. *Medical Journal of Australia* 194: 65-7.
6. Bryszewska, M. A., L. Tomas-Cobos, E. Gallego, M. P. Villalba, D. Rivera, D. L. Taneyo Saa, A. Gianotti. 2019. In vitro bioaccessibility and bioavailability of iron from breads fortified with microencapsulated iron. *LWT - Food Science and Technology* 99: 431-437.
7. Buzzo, M. L., F. M. H. Carvalho, P. Tiglea, L. J. de Arauz, E. E. K. Arakaki, and R. Matsuzaki. 2012. Monitoring the wheat and corn flours enriched with iron. *Revista do Instituto Adolfo Lutz* 71: 645-649.
8. Chiplonkar, S. A., V. V. Agte, K. V. Tarwadi, and R. Kavadia. 1999. In vitro dialyzability using meal approach as an index for zinc and iron absorption in humans. *Biological Trace Element Research* 67: 249-256.
9. Cortes, F., C. Mellado, and R. A. Pardo. 2012. Wheat flour fortification with folic acid: changes in neural tube defects rates in Chile. *American Journal of Genetics* 158A: 1885-90.
10. Field, M. S., P. Mithra,   D. Estevez,   and J. P. Pena-Rosas. 2020. Wheat flour fortification with iron for reducing anaemia and improving iron status in populations. Cochrane Database of Systematic Reviews, 7, Art. No.: CD011302. Accessed 30 August 2021.
11. Gujska, E., and K. Majewska. 2005. Effect of baking process on added folic acid and endogenous folates stability in wheat and rye breads. *Plant Foods for Human Nutrition* 60: 37-42.
12. Nestel, P., R. Nalubola, R. Sivakaneshan, A. R. Wickramasinghe, S. Atukorala, and T. Wickramanayake. 2004. The use of iron-fortified wheat flour to reduce anemia among the estate population in Sri Lanka. *International* *Journal for Vitamin and Nutrition Research* 74 (1): 35-51.
13. Noor, R. A, A. I., Abioye, N., Ulenga, S., Msham, G., Kaishozi, N. S., Gunaratna, [R. Mwiru](https://pubmed.ncbi.nlm.nih.gov/?term=Mwiru+R&cauthor_id=28797054), [E. Smith](https://pubmed.ncbi.nlm.nih.gov/?term=Smith+E&cauthor_id=28797054), [C. N. Dhillon](https://pubmed.ncbi.nlm.nih.gov/?term=Dhillon+CN&cauthor_id=28797054), [D. Spiegelman](https://pubmed.ncbi.nlm.nih.gov/?term=Spiegelman+D&cauthor_id=28797054).  et al. 2017. Large scale wheat flour folic acid fortification program increases plasma folate levels among women of reproductive age in urban Tanzania. *PLoS ONE* 12 (8): e0182099.
14. Pachón, H., R. J. Stoltzfus, and R. P. Glahn. 2008. Chicken thigh, chicken liver, and ironfortified wheat flour increase iron uptake in an in vitro digestion/Caco-2 cell model. *Nutrition Research* 28: 851-858.
15. Rebellato, A. P., B. Klein, R. Wagner, and J. A. L. Pallone. 2018. Fortification effects of different iron compounds on refined wheat flour stability. *Journal of Cereal Science* 82: 1-7.
16. Rebellato, A. P., J. Bussi, J. G. S. Silva, R. Greiner, C. J. Steel, and J. A. L. Pallone. 2017. Effect of different iron compounds on rheological and technological parameters as well as bioaccessibility of minerals in whole wheat bread. *Food Research International* 94: 65-71.
17. Sadighi, J., R. Sheikholeslam, K. Mohammad, H. Pouraram, Z. Abdollahi, K. Samadpour, [F. Kolahdooz](https://pubmed.ncbi.nlm.nih.gov/?term=Kolahdooz+F&cauthor_id=17645902), and [M. Naghavi](https://pubmed.ncbi.nlm.nih.gov/?term=Naghavi+M&cauthor_id=17645902). 2008. Flour fortification with iron: A mid-term evaluation. *Public Health* 122: 313-321.
18. Skrbic, B., S. Milovac, D. Dodig, and B. Filipcev. 2009. Effects of hull-less barley flour and flakes on bread nutritional composition and sensory properties. *Food Chemistry* 115: 982-988.
