# Supplementary material for: Critical assessment of wheat biofortification for iron and zinc: a comprehensive review of conceptualization, trends, approaches, bioavailability, health impact, and policy framework
Source: Front Nutr. 2024 Jan 4;10:1310020. doi: 10.3389/fnut.2023.1310020 (PMC10794668; doi:10.3389/fnut.2023.1310020)
Supplement: Supplementary file 4 [file Table_4.DOCX]

**Table S4:** Advantages and disadvantages of food fortification.

| **Advantage** | **Disadvantage** |
| --- | --- |
| Regular intake of fortified foods shall meet the nutritional requirements more efficiently and effectively than will intermittent supplements | Due to increase in selected micronutrients, it is not a substitute for a good quality diet that supplies adequate amounts of energy, protein, essential fats and other food constituents. |
| It helps in ameliorating the risk of the multiple micronutrient deficiency especially in growing children and women of fertile age that would otherwise result from seasonal deficits in the food supply or a poor quality diet. | A specific fortified foodstuff might not be consumed by all members of a target population. For example, it is difficult to find appropriate food to fortify in population relying on local or domestic production of rice and maize. |
| Excellent way of increasing the vitamins in breast milk reducing the need for supplementation in postpartum women and infants. | Everyone in the population is exposed to increased levels of micronutrients in food, irrespective of whether or not they will benefit from fortification. |
| It has great potential to improve the nutritional status of a large proportion of the population, both poor and wealthy in developing countries consuming industry-processed, rather than locally-produced, foods. | Technological issues relating to food fortification have yet to be fully resolved, especially with regard to appropriate levels of nutrients, stability of fortificants, nutrient interactions, physical properties, as well as acceptability by consumers including cooking properties and taste. |
| It requires neither changes in existing food patterns nor individual compliance. | Infants and young children, who consume relatively small amounts of food, are less likely to be able to obtain their recommended intakes of all micronutrients from universally fortified staples. |
| Fortification is often more cost-effective than other strategies, especially if the technology already exists and if an appropriate food distribution system is in place | Fortified foods often fail to reach the poorest segments of the general population who are at the greatest risk of micronutrient deficiency due to low purchasing power and an underdeveloped distribution channel. |
