# Supplementary material for: Critical assessment of wheat biofortification for iron and zinc: a comprehensive review of conceptualization, trends, approaches, bioavailability, health impact, and policy framework
Source: Front Nutr. 2024 Jan 4;10:1310020. doi: 10.3389/fnut.2023.1310020 (PMC10794668; doi:10.3389/fnut.2023.1310020)
Supplement: Supplementary file 5 [file Table_5.DOCX]

**Table S5:** Methods for estimating Fe and Zn bioavailability *in vitro* and *in vivo.*

| **Methods** | **Description** | **Scope** |
| --- | --- | --- |
| ***In vitro* bioaccesibility methods** | | |
| Gastro intestinal models | **Solubility:** It measures the fraction of minerals released from the ingested food materials and is available for absorption. | **Merit:** Easy to perform as it requires minimum equipment and is cost effective  **Limitation:** Cannot measure rate of nutrient uptake, absorption and transport. |
|  | **Dialyzability:** It measures the fraction of soluble minerals that can cross the dialysis membrane of certain molecular weight cut off and is available for passive diffusion through mucosa |  |
| TNO’s intestinal model (TIM) | This is computer controlled sophisticated model since many parameters of human digestion system *viz.* body temperature, flow of saliva, gastric and pancreatic juice, peristalsis and churning, regulation of pH *etc.* are simulated | **Merit:** As it includes many parameters hence allows collection of digest at any step of the digestion.  **Limitation:** Expensive and limited validation studies. |
| ***In vitro* bioavailability methods** | | |
| Caco-2 cell model | This cell belong to human epithelial cell line derived from colonic adenocarcinoma and is extensively used in combination with gastrointestinal model | Allows the study of nutrient component competition at site of absorption however it requires trained personal with knowledge of cell culture methods |
| ***In vivo* bioavailability methods** | | |
| Animal models | Provide information about in vivo bioavailability, uses several animals like mice and rats, Gerbils, ruminant calf, ferrets etc. | Allows whole body assessment of nutrient absorption by dissecting and analysis of individual tissues,  **Limitation:** despite several appropriate animal model, no one can exactly simulate the physiological response of human |
| Human studies | Provide the most applicable results as it consider host factor, disease, physiology etc. | Allows use of radioactive and stable isotopes in human studies for discrimination between the dose of micronutrient provided and endogenous level allowing more accurate measurement of bioavailability. |
| Mathematical model | Algorithm are used to predict Fe/Zn bioavailability | It is dependent on accurate information on food intake and dietary absorption modifiers |
