# Supplementary figures and images for: Critical assessment of wheat biofortification for iron and zinc: a comprehensive review of conceptualization, trends, approaches, bioavailability, health impact, and policy framework
Source: Front Nutr. 2024 Jan 4;10:1310020. doi: 10.3389/fnut.2023.1310020 (PMC10794668; doi:10.3389/fnut.2023.1310020)

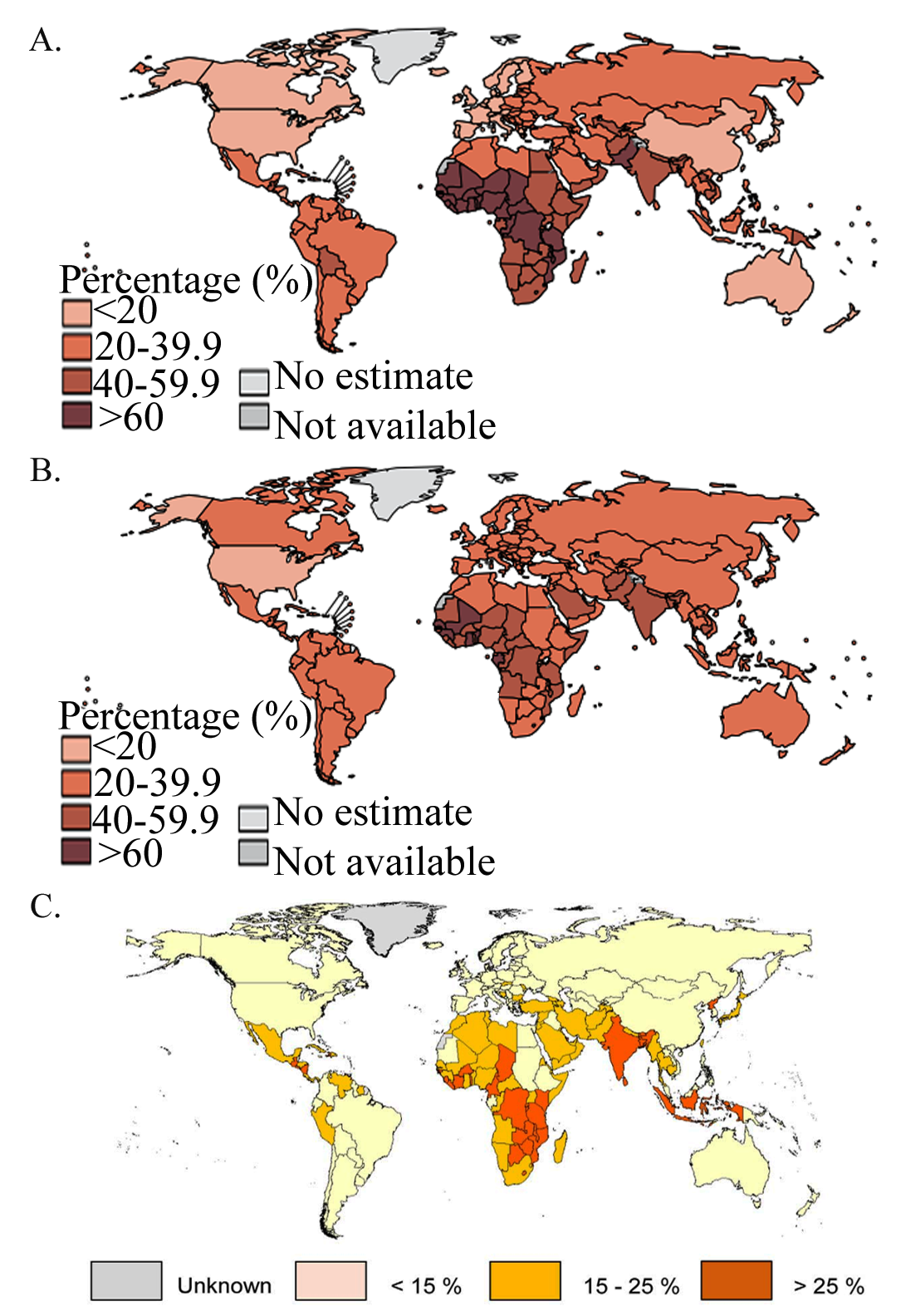

Supplement: Supplementary file 6 [file Image_1.TIF]
